# Medicinal Plants and Phytochemicals in Cardioprotection—Mechanistic Pathways and Translational Roadmap

**DOI:** 10.3390/life16010175

**Published:** 2026-01-21

**Authors:** Diana Maria Morariu-Briciu, Alex-Robert Jîjie, Sorin Lucian Bolintineanu, Ana-Maria Pah, Sorin Dan Chiriac, Adelina Chevereșan, Victor Dumitrașcu, Cătălin Prodan Bărbulescu, Radu Jipa

**Affiliations:** 1 Department of Anatomy and Embryology, Faculty of Medicine, “Victor Babeş” University of Medicine and Pharmacy, 300041 Timisoara, Romania; diana-maria.morariu-briciu@umft.ro (D.M.M.-B.); s.bolintineanu@umft.ro (S.L.B.); catalin.prodan-barbulescu@umft.ro (C.P.B.); 2 Doctoral School, “Victor Babeş” University of Medicine and Pharmacy, 300041 Timisoara, Romania; 3 Department of Toxicology, Drug Industry, Marketing, Management, and Dermatopharmacy, Faculty of Pharmacy, “Victor Babeş” University of Medicine and Pharmacy, 300041 Timisoara, Romania; alex-robert.jijie@umft.ro; 4 Research Center for Pharmaco-Toxicological Evaluations, “Victor Babeş” University of Medicine and Pharmacy, 300041 Timisoara, Romania; 5 Cardiology Department, Faculty of Pharmacy, “Victor Babeş” University of Medicine and Pharmacy, 300041 Timisoara, Romania; 6 Department of Surgery II, Faculty of Medicine, “Victor Babeş” University of Medicine and Pharmacy, 300041 Timisoara, Romania; 7 Department of Pharmacology, Faculty of Medicine, “Victor Babeş” University of Medicine and Pharmacy, 300041 Timisoara, Romania; cheveresan.adelina@umft.ro (A.C.); dumitrascu.victor@umft.ro (V.D.); 8 2nd Surgery Clinic, Timisoara Emergency County Hospital, 300723 Timisoara, Romania; 9 Department of “Life Science”, Faculty of Medicine, “Vasile Goldiş” Western University of Arad, 310048 Arad, Romania; jipa.radu@uvvg.ro

**Keywords:** cardioprotection, cardioprotective plants, cardioprotective phytochemicals, herbal medicine

## Abstract

Despite major advances in guideline-directed cardiovascular therapy, residual cardiovascular risk persists, partly driven by oxidative stress, chronic inflammation, endothelial dysfunction, and mitochondrial injury not fully addressed by current drugs. Translation of plant-based cardioprotectants is constrained by preparation-dependent variability in extract chemistry (plant part/cultivar/processing and extraction method), low and variable systemic exposure for key actives (notably curcuminoids and many polyphenols), and clinically relevant safety/interaction considerations (e.g., hepatotoxicity reports with concentrated green tea extracts and antiplatelet-related bleeding-risk considerations for some botanicals). We therefore provide a mechanism- and translation-oriented synthesis of evidence for cardioprotective botanicals, chosen for long-standing traditional use and scientific validation with reproducible experimental data and, where available, human studies, including *Crataegus monogyna*, *Allium sativum*, *Olea europaea*, *Ginkgo biloba*, *Leonurus cardiaca*, and *Melissa officinalis*. Across studies, polyphenols (especially flavonoids and phenolic acids) and organosulfur compounds are most consistently associated with cardioprotection, while terpene-derived constituents and secoiridoids contribute mechanistically in plant-specific settings (e.g., *Ginkgo* and *Olea*). Predominantly in experimental models, these agents engage redox-adaptive (Nrf2), mitochondrial (mPTP), endothelial, and inflammatory (NF-κB) pathways, with reported reductions in ischemia–reperfusion injury, oxidative damage, and apoptosis. Clinical evidence remains heterogeneous and is largely confined to short-term studies and surrogate outcomes (blood pressure, lipids, oxidative biomarkers, endothelial function), with scarce data on hard cardiovascular endpoints or event reduction. Priorities include standardized, chemotype-controlled formulations with PK/PD-guided dosing and adequately powered randomized trials that assess safety and herb–drug interactions.

## 1. Introduction

Cardiovascular diseases (CVDs) remain the leading cause of morbidity and mortality worldwide, with prevalence further amplified by population ageing and cardiometabolic risk factors such as obesity and type 2 diabetes [1,2,3,4]. This sustained burden, together with substantial healthcare and productivity costs and persistent inequities in access to preventive and advanced care, underscores the need for effective and scalable strategies that complement contemporary cardiovascular management [5].

The contemporary management of atherosclerotic and other major cardiovascular conditions involves a comprehensive approach that includes population-level prevention, lifestyle modifications, and evidence-based pharmacotherapy (e.g., lipid-lowering agents, antiplatelet drugs, antihypertensives) alongside invasive procedures when warranted [2,6]. Although these strategies have reduced cardiovascular mortality and improved outcomes, their real-world effectiveness is persistently constrained by adverse effects, limited access, and suboptimal long-term adherence [5,7]. Even with guideline-directed therapies, many patients remain at significant residual cardiovascular risk, including residual inflammatory risk (e.g., persistently elevated hsCRP), residual lipid-related risk (e.g., triglyceride-rich lipoproteins and lipoprotein (a) despite optimized LDL-C lowering), residual thrombotic risk, and cardiometabolic risk driven by diabetes/obesity and endothelial dysfunction, pathological drivers that are incompletely addressed by current treatments [2,6,7]. Furthermore, long-term therapy can be limited by a trade-off between efficacy, tolerability, access, and adherence, which may reduce real-world effectiveness [8]. Consequently, there is an increasing interest in complementary and alternative therapies, including nutraceuticals and herbal preparations, which may extend treatment options for cardioprotection through diverse mechanisms and improved safety profiles [5,6,9].

Plant-derived bioactive compounds are secondary metabolites produced by plants known for their biological activity. Historically utilized in traditional medicine and increasingly explored as potential therapeutic agents, these compounds encompass a range of classes relevant to cardiovascular health, including polyphenols (e.g., flavonoids, phenolic acids), alkaloids, terpenoids, saponins, and others [10,11,12,13]. Phytochemicals may be administered as isolated compounds (e.g., curcumin, resveratrol, quercetin) or as complex plant extracts, both of which have shown cardioprotective effects predominantly in preclinical settings, with a subset progressing to clinical investigation. This distinction is critical for reproducibility and translation: isolated compounds enable defined purity, dosing, and PK/PD characterization, whereas extracts exhibit batch-to-batch variability driven by chemotype, cultivation/harvesting conditions, and processing, which can confound attribution of efficacy and safety to specific constituents. Conversely, extracts may provide multi-target activity through additive or synergistic interactions, but require rigorous standardization and quality control to support mechanistic interpretation, regulatory acceptance, and reliable clinical evaluation [14,15,16,17,18].

A distinguishing characteristic of many plant-derived cardioprotective agents, as suggested largely by preclinical research, is their multimodal action. Rather than acting through a single target, phytochemicals have been reported, predominantly in in vitro, ex vivo, and animal models, to influence multiple pathways crucial to cardiovascular disease pathogenesis, including oxidative stress, inflammation, and endothelial dysfunction [18,19,20]. For instance, polyphenols have been shown in experimental systems to reduce reactive oxygen species (ROS) and enhance cytoprotective signaling, thereby limiting oxidative damage in cardiac and vascular cells [21]. Concurrent anti-inflammatory actions observed mainly in preclinical models further support cardiovascular protection by attenuating chronic vascular inflammation [19]. In models of myocardial injury, selected phytochemicals have been reported to exert anti-apoptotic effects and to modulate autophagy-related pathways, with relevance to ischemic injury and drug-induced cardiotoxicity [18]. Likewise, evidence from cellular and animal ischemia models suggests that specific saponins can promote endothelial protection and pro-angiogenic signaling, potentially supporting vascular repair. However, the clinical relevance and translatability of these mechanisms remain to be established [22,23]. Beyond direct myocardial and vascular mechanisms, cardioprotection may also be achieved indirectly by modulating upstream cardiometabolic risk factors that drive disease progression. Accordingly, several phytochemicals have been reported (predominantly in preclinical and early clinical studies) to influence systemic metabolic determinants of CVD, including lipid homeostasis and related dysmetabolic profiles [4,23,24].

An increasing body of evidence supports the protective roles of phytochemicals across a range of cardiovascular conditions, with numerous in vivo and in vitro studies demonstrating efficacy against various models of myocardial injury and thrombotic events [7,17,19,20,25]. Some plant-derived agents have progressed to clinical trials, where they have shown potential benefits at the population level, emphasizing the need for well-designed randomized trials with uniform protocols and meaningful clinical outcomes [1,9]. However, substantial translational challenges persist, including issues related to variable bioavailability, instability, and the rapid metabolism of many natural compounds, as well as variability and inconsistency in botanical extracts [8,14,26]. Strategies to enhance clinical applicability involve formulation science techniques, rigorous standardization of phytochemicals, and advanced pharmacological methodologies to better understand and prioritize therapeutic prospects [14,27,28,29].

In light of the compositional variability of botanical preparations, heterogeneity of experimental designs, and the limited and often inconsistent clinical evidence, together with key translational uncertainties related to standardization and bioavailability, the present review aims to (1) synthesize and contextualize the principal mechanisms of plant-derived cardioprotection; (2) highlight plant sources and phytochemicals supported by the most reproducible experimental evidence and, where available, human data; (3) critically appraise preclinical and clinical findings with attention to methodological limitations and outcome relevance; and (4) define priority research directions to improve translatability, including extract standardization/chemotype control, PK/PD-guided optimization of bioavailability, and rational evaluation of combinations with guideline-directed therapies.

## 2. Concept of Cardioprotection in Cardiovascular Research

Cardioprotection refers to the ensemble of endogenous adaptive responses and exogenous interventions that reduce myocardial injury and preserve cardiac structure and function in the face of acute or chronic stressors, most notably ischemia/reperfusion (I/R), as well as pharmacologic cardiotoxins and surgical insults. Over the past decades, the field has adopted outcome-oriented definitions in which the success of a cardioprotective maneuver is judged by reductions in tissue necrosis and cell death, preservation of contractile performance and electrical stability, and mitigation of adverse remodeling. These outcomes have been operationalized in preclinical and clinical studies as infarct size, biochemical biomarkers of myocyte injury, imaging-derived salvage and functional parameters, and clinical endpoints, including heart failure and death [30,31,32,33].

The contemporary framework of cardioprotection was catalyzed by ischemic preconditioning (IPC), in which brief sublethal ischemia activates endogenous signaling that increases myocardial tolerance to subsequent ischemia/reperfusion injury [34]. IPC quickly catalyzed a large mechanistic enterprise that identified triggers (adenosine, bradykinin, nitric oxide), early mediators (reactive oxygen species—ROS), ionic effectors (sarcolemmal and mitochondrial ATP sensitive K^+^ channels, KATP), and downstream signal transduction modules (protein kinase C isoforms, PI3K–Akt–ERK prosurvival kinases, and regulators of the mitochondrial permeability transition pore, mPTP) as central components of the cardioprotective cascade. These discoveries reframed cardioprotection from a descriptive phenomenon to a mechanistically tractable therapeutic concept [34,35,36]. In parallel, pharmacological agents (e.g., volatile anesthetics, opioids, KATP modulators, Toll-like receptor ligands) were shown to recapitulate core features of IPC (pharmacological or anesthetic preconditioning), demonstrating that molecular pathways of cardioprotection are amenable to drug-based manipulation and thereby accelerating translational interest [37,38,39,40].

Subsequent expansions of the paradigm included delayed (second window) preconditioning, ischemic postconditioning (brief cycles of reperfusion/ischemia at the onset of reperfusion), and remote ischemic conditioning (brief ischemia–reperfusion applied to a limb or distant organ producing cardioprotection), each of which extended the operational definition of cardioprotection and highlighted systemic neurohumoral as well as local myocardial mechanisms [32,41,42,43]. Importantly, these conceptual advances created a translational pipeline in which experimental manipulations (e.g., mPTP blockade by cyclosporine A) could be tested in patients with acute myocardial infarction. However, translation has proved challenging and underscores the complexity of clinical cardioprotection [33,44].

**Figure 1:**
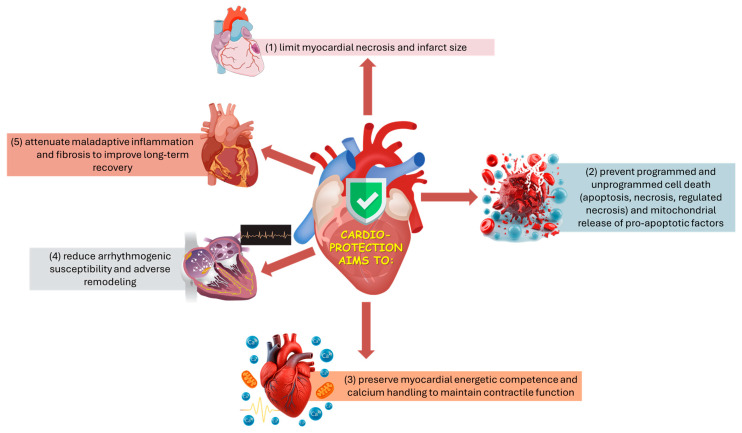
Schematic overview of the main targets of cardioprotection.

From an applied biomedical perspective, cardioprotection aims to limit infarct development and preserve post-injury cardiac performance by coordinating control of mitochondrial permeability and energy/Ca^2+^ homeostasis, suppression of regulated cell-death programs, and modulation of arrhythmogenic, inflammatory, and fibrotic remodeling processes (Figure 1) [30,31,33,34]. Because these processes are tightly coupled, stabilizing mitochondria, particularly by preventing mPTP opening, can simultaneously reduce necrotic cell death and downstream inflammatory amplification, improving infarct size and post-ischemic function [39,45].

From a mechanistic standpoint, cardioprotective strategies converge on a set of recurrent cellular targets and processes that together determine myocardial fate during and after ischemia:

- Mitochondria as nodal effectors:

Mitochondria occupy a central position in cardioprotection by integrating metabolic, redox, and cell-death signals, and by shaping susceptibility to reperfusion injury. Inhibition of mPTP opening at reperfusion is widely recognized as a final common effector of multiple conditioning strategies [39,40,45]. Additional mitochondrial-associated mechanisms implicated in protection include activation of mitochondrial KATP channels (mitoKATP) and maintenance of hexokinase II (HKII) association with the outer mitochondrial membrane, both of which have been linked to improved mitochondrial stability and reduced pro-death signaling during reperfusion [37,46,47,48]. Moreover, conditioned myocardium recruits mitochondrial quality control processes (mitophagy and regulated dynamics of fission/fusion) that remove dysfunctional mitochondria and thereby enhance post-ischemic recovery [49,50].

- Redox signaling and reactive oxygen species:

ROS play an important, double-edged role in cardioprotection: low to moderate ROS bursts act as necessary signaling triggers for IPC and for several pharmacological preconditioning mimetics, whereas excessive or sustained oxidative stress mediates cellular injury during reperfusion. Accordingly, experimental studies have shown that ROS scavengers can abrogate preconditioning in some models, and ROS generators (e.g., menadione) can mimic IPC when applied at sublethal doses. The net effect depends on timing, subcellular localization (mitochondrial versus cytosolic), and the balance between ROS formation and antioxidant buffering capacity [51,52,53,54].

- Ion channels and calcium homeostasis:

Preservation of calcium homeostasis is fundamental to cardioprotection: preconditioning attenuates reperfusion-induced calcium overload, and manipulation of ion channels—including sarcolemmal and mitochondrial KATP channels—modulates the myocardium’s resistance to injury; pharmacologic blockade of KATP channels abolishes IPC in several preparations, demonstrating their causal role in many models [55,56,57,58]. Calcium signaling also interfaces with other modules (e.g., IP3 pathways, PKC activation) that influence survival versus death decisions [59].

- Prosurvival kinase cascades and phosphorylation networks:

Conditioning stimuli activate prosurvival kinase programs—commonly summarized as the reperfusion injury salvage kinase (RISK) pathway (PI3K–Akt, ERK1/2) and other modulators including PKC isoforms (notably PKCε) and GSK 3β—that converge on mitochondrial targets (including mPTP regulation) and on transcriptional responses; phosphorylation of Akt and ERK at reperfusion is a reproducible molecular signature of IPC-mediated protection in experimental hearts [37,38,40,60]. PKCε translocation to mitochondria and phosphorylation of mitochondrial substrates (e.g., connexin43) have been implicated in the preservation of mitochondrial function and in limiting apoptotic signaling [37,60].

- Metabolic and transcriptional reprogramming:

Delayed (second-window) preconditioning and some pharmacologic interventions extend beyond acute post-translational signaling by engaging longer-term metabolic and transcriptional adaptation programs that increase stress resistance. These programs can upregulate antioxidant defenses and electron transport chain components and remodel substrate utilization, thereby improving tolerance to subsequent ischemic or metabolic insults [61,62]. Transcriptional induction of nuclear-encoded mitochondrial genes after delayed IPC illustrates this genomic dimension of cardioprotection [61,62]. In parallel, nutrient-sensing regulators such as TXNIP and thioredoxin systems interface with mitochondrial function and redox balance, linking metabolic status to cardioprotective resilience [63].

- Inflammation, complement, and extracellular mediators:

Conditioning influences local and systemic inflammatory responses: IPC reduces myocardial complement gene expression and modulates cytokine pathways, while systemic inflammatory mediators (and transcription factors such as NF κB) participate in the heart’s response to surgical or ischemic stress. Manipulation of inflammatory signaling represents both a target and a readout of cardioprotection [64,65].

Experimental assessment of cardioprotection employs a hierarchy of models and endpoints that span reductionist preparations to animal and human studies. In preclinical laboratories, infarct size (commonly measured by 2,3,5 triphenyl tetrazolium chloride, TTC, staining in experimental ischemia models) remains the canonical metric of tissue salvage, often complemented by measures of mitochondrial function, cytochrome C release, ROS production, and markers of apoptosis and necrosis [37,45,64]. Isolated perfused heart preparations and single cell myocyte studies enable mechanistic dissection (e.g., KATP channel function, PKCε translocation to mitochondria, mitoKATP opening) that link molecular events to functional outcomes [37,66,67,68]. In translational and clinical research, biochemical biomarkers (cardiac troponins), imaging modalities (cardiac MRI, nuclear perfusion imaging), and hard clinical endpoints (mortality, heart failure) are used to quantify protection and to evaluate therapeutic candidates, but differences between experimental conditions and clinical heterogeneity have complicated translation [31,32,33].

Several pharmacological and natural interventions have been instrumental in defining and dissecting cardioprotective mechanisms. Classical humoral mediators of IPC (adenosine, bradykinin, and nitric oxide) were among the earliest triggers implicated in initiating protection and have been studied in both experimental and clinical contexts [35,36]. Mechanistically, these mediators activate receptor- and endothelium-linked signaling that engages kinase cascades and NO-dependent pathways, which converge on mitochondrial effectors (including KATP channel- and mPTP-related processes) to increase tolerance to ischemia/reperfusion injury [35,36]. Volatile anesthetics (e.g., isoflurane) and opioids (e.g., morphine) produce anesthetic- and opioid induced preconditioning that reproduces many IPC phenotypes and clarified intracellular effectors such as KATP channels and PKC isoforms [41,42,43,66]. Experimental application of ROS-generating agents at sublethal doses (e.g., menadione) mimicked IPC, substantiating the role of regulated ROS bursts as triggers [69], while free radical scavengers could negate protection in some models, underscoring the finely balanced role of redox signaling [70,71]. Natural products and botanical extracts (e.g., garlic preparations) have been reported to elicit IPC-like cardioprotective effects predominantly in preclinical models, supporting the concept that phytochemical agents can engage conserved endogenous defense programs [72]. Importantly, these effects often converge on canonical cardioprotective nodes described for conditioning stimuli, including redox-adaptive signaling, endothelial mediator pathways (e.g., NO/H_2_S), kinase-driven cytoprotective cascades, and mitochondrial stabilization with downstream control of mPTP opening. This mechanistic convergence provides a unifying rationale for evaluating botanicals as multi-target adjuncts that may address residual risk processes insufficiently mitigated by single-pathway therapies, while also highlighting the need for rigorous standardization and PK/PD-informed dosing to improve translatability. At the molecular therapeutic level, blockade of mPTP opening by cyclosporine A entered clinical testing on the premise that inhibiting mPTP at reperfusion would reduce infarct size, exemplifying how mechanistic insight can motivate early clinical translation [39,44].

Emerging evidence indicates that cardioprotective efficacy is strongly modulated by comorbid conditions (e.g., diabetes, ageing, and sex hormone status), concomitant medications (e.g., P2Y12 inhibitors and anesthetics), and the timing and intensity of the conditioning stimulus. These factors can markedly attenuate, reshape, or mask cardioprotective signaling, such that benefits observed in reductionist models may not be reproducible across heterogeneous, poly-medicated clinical populations [44,73,74,75]. Remote ischemic conditioning, which appeared promising in mechanistic and pilot clinical studies, produced neutral results in a large randomized trial of ST elevation myocardial infarction (CONDI 2/ERIC PPCI), emphasizing the challenges of clinical translation and the need to reconcile heterogeneity between model systems and patient cohorts [33,43,73,76]. Further complicating translation, conditioning stimuli are not universally benign: conditioning responses are tissue-, cell type-, and context-dependent and, in non-cardiac models, can even exacerbate injury under specific experimental conditions (e.g., deleterious effects on oligodendrocytes reported in some cerebral preconditioning paradigms) [32,77]. Collectively, these observations underscore that cardioprotective strategies must be calibrated to biological context and evaluated with explicit attention to safety, dose-timing parameters, and patient-level heterogeneity [32,77].

Collectively, decades of experimental and translational research indicate that cardioprotection converges on a conserved set of mechanistic nodes, including mitochondrial stability (notably regulation of mPTP opening and related mitochondrial effectors), redox and ion/Ca^2+^ homeostasis, pro-survival kinase signaling, mitochondrial quality control, and regulated inflammation, that shape myocardial resilience to ischemic and non-ischemic insults [38,39,40,45,49,50]. This mechanistic framework has informed targeted strategies (e.g., mPTP inhibition, MQC- and kinase-directed interventions, and remote conditioning paradigms) and provides a direct rationale for exploring phytochemicals and botanical extracts as multi-target modulators capable of engaging these same endogenous protective pathways [44,49,72,78]. However, the recurrent mismatch between experimental promise and clinical outcomes underscores the need for rigorous translational frameworks that explicitly account for comorbidity, drug–drug/herb–drug interactions, timing, and clinically meaningful endpoints. Addressing these challenges remains essential if cardioprotective biology is to yield robust therapies that improve patient outcomes [31,32,33,73].

## 3. Common Cardioprotective Plants

In this section, we synthesize evidence for ten medicinal plant species with long-standing cardiovascular use that are also supported by reproducible experimental findings and, where available, human data. Rather than providing exhaustive monographs, we emphasize mechanistic interpretation and translational readiness by summarizing (i) the dominant phytochemical classes and standardized preparations, (ii) the convergent cardioprotective pathways mapped to the conserved nodes discussed above (e.g., redox/inflammatory control, endothelial function, and mitochondrial stability), and (iii) key limitations affecting clinical reproducibility, including extract variability, bioavailability, and safety in polypharmacy contexts.

### 3.1. Crataegus monogyna *Jacq.* (Hawthorn)

#### 3.1.1. Historical Background

*Crataegus monogyna* Jacq. (hawthorn, Rosaceae family) is a well-established European cardiotonic botanical with long-standing use for functional cardiovascular complaints (e.g., palpitations and mild hypertension) and substantial pharmacological evaluation supporting this traditional rationale [79,80,81]. Ethnobotanical surveys report broadly consistent use across Mediterranean and Central European regions, with infusions or preparations from flowers, leaves, and fruits employed for hypertension, palpitations, and related circulatory symptoms, including in Romania [82,83,84]. Importantly for translation, European pharmacopoeias recognize hydroalcoholic extracts of leaves and flowering tops, and standardized preparations (notably WS^®^1442) have progressed into clinical testing, enabling more reproducible assessment of efficacy and safety than non-standardized products [79,81,85]. Related *Crataegus* species are used in other medical systems (e.g., *C. pinnatifida* in East Asia), underscoring cross-cultural convergence on hawthorn-derived products for cardiovascular indications [86,87,88].

#### 3.1.2. Relevant Cardioprotective Phytocompounds

The cardioprotective properties of *Crataegus monogyna* have been attributed to a complex phytochemical matrix. Phytochemical profiling of *C. monogyna* consistently identifies polyphenols as the dominant bioactive fraction, with flavonoids (flavonol glycosides and related aglycones) and phenolic acids/anthocyanins most frequently implicated in the antioxidant, vasoregulatory, and endothelial effects attributed to hawthorn preparations [79,86,87,88,89,90]. These polyphenolic constituents are distributed across leaves, flowers, and fruits, with anthocyanin enrichment in ripe berries supporting additional lipid-modulating and endothelial-relevant activities [87,90]. Beyond polyphenols, triterpene/sterol-like fractions (e.g., cycloartenol-rich constituents) and minor components such as saponins and essential oils have been reported and provide complementary anti-inflammatory and functional contributions, although evidence for these classes is generally less extensive and more preparation-dependent [80,83,87,89,91].

Critically, the relative abundance of these phytochemical groups varies substantially with plant organ (leaves/flowers vs. berries), geographic origin, and post-harvest processing, and extraction conditions can markedly shift the recovered chemical profile and apparent bioactivity. Solvent selection is therefore a major determinant of reproducibility: in some endothelial assays, water fractions showed limited activity, whereas ethanol/methanol/acetone extracts retained bioactive fractions, underscoring the need to report and standardize extraction parameters when comparing studies or translating findings [83,88,92].

#### 3.1.3. Mechanisms of Cardioprotective Effects and Medicinal Benefits

Collective experimental and clinical literature supports a multi-modal cardioprotective profile for *Crataegus monogyna*, in which different phytochemical fractions engage complementary molecular and physiological mechanisms. The principal mechanisms, with types of evidence, are summarized below:

- Antioxidant and direct ROS scavenging:

Hawthorn extracts and isolated polyphenolic fractions have shown robust radical-scavenging and lipid peroxidation–inhibiting properties in chemical assays and in vitro model systems. Electron paramagnetic resonance and biochemical studies indicate scavenging of superoxide-, hydroxyl-, and peroxyl-type radicals by *Crataegus* preparations, alongside reductions in lipid peroxidation and myoglobin oxidation in experimental settings [88,90,93]. In vivo, mainly in rodent models of cardiac injury (e.g., doxorubicin-induced chronic heart failure), hawthorn treatment has been associated with lower markers of oxidative injury (e.g., malondialdehyde) and increased endogenous antioxidant enzyme activity (e.g., glutathione peroxidase, catalase) [84,94]. While these findings support antioxidant/ROS-modulatory potential, the extent to which they translate into clinically meaningful cardioprotection remains incompletely established.

- Anti-inflammatory signaling and cytokine modulation:

Anti-inflammatory effects of hawthorn constituents have been reported primarily in experimental models. For example, a triterpene fraction reduced leukocyte migration and inhibited phospholipase A2 activity in acute inflammation paradigms, suggesting modulation of early inflammatory cascades [91]. In animal models of cardiac injury, hawthorn extracts have been associated with reduced myocardial pro-inflammatory cytokine expression (e.g., IL-1β, TNF-α) and attenuated histological inflammation, paralleling functional improvements in preclinical settings [84,94,95]. However, confirmatory clinical data for anti-inflammatory endpoints in cardiovascular populations remain limited.

- Endothelial protection, barrier integrity, and NO-mediated vasodilation:

Evidence from endothelial/vascular experimental models and limited clinical studies suggests that hawthorn preparations can improve endothelial function. An example of clinical evidence (surrogate endpoint) is a randomized, controlled crossover trial that reported that a standardized hawthorn extract improved flow-mediated dilation (FMD; an NO-dependent surrogate measure of endothelial function) in adults with prehypertension or mild hypertension [96]. Fractionation and molecular studies of WS^®^1442 indicate dual endothelial actions: promotion of barrier integrity via cortactin activation and inhibition of barrier-disruptive calcium signaling. These effects were localized to non-aqueous phytochemical fractions (ethanol/methanol/acetone) rather than the water fraction [92,97]. Together, these data support both vasodilatory (NO-mediated) and barrier-stabilizing effects of hawthorn phytochemicals (predominantly polyphenolic fractions) in clinical and laboratory settings [92,96,97].

- Lipid-lowering and anti-atherosclerotic effects

Hawthorn fruit and leaf extracts have shown hypolipidemic activity in animal models of dyslipidemia and cardiometabolic disease (e.g., JCR:LA-cp rodents) with improved plasma lipid profiles and concurrent cardiac benefits, supporting an anti-atherogenic potential of hawthorn polyphenols [90,98]. Studies concerning *Crataegus* clinical usage report favorable effects on hyperlipidemia and cardiovascular risk factors in clinical studies, although heterogeneity in extracts and study designs limits quantitative conclusions [88,99].

- Anti-apoptotic, anti-ischemia/reperfusion, and mitochondrial-protective actions:

Hawthorn extracts reduce ischemia–reperfusion (I/R) injury and biochemical indices of myocardial necrosis in perfused heart and cellular models. These cardioprotective outcomes are associated with ROS scavenging, preserved energy turnover, and reduced release of myocardial enzymes (CK, LDH) in preclinical experiments [93,94,100]. Although direct molecular dissection in hawthorn preparations is incomplete, these effects are consistent with established cardioprotective strategies that inhibit mitochondrial pathways of cell death [101,102].

- Positive inotropy and anti-arrhythmic properties:

In isolated human cardiac tissue and other experimental systems, standardized *Crataegus* extracts produced concentration-dependent increases in myocardial contractility accompanied by transient intracellular calcium changes. Studies suggest a cAMP-independent inhibition of Na^+^/K^+^-ATPase and concomitant improvements in myocyte energy turnover that differ from classical cardiac glycosides [103]. Additionally, *Crataegus* extracts can prolong action potential duration and refractory period, yielding anti-arrhythmic effects observed in experimental studies and invoked in clinical usage as cardiotonic/antiarrhythmic agents [99,103,104].

- Pro-cardiogenic and pro-angiogenic cues from stem/progenitor populations:

Recent studies indicate that WS^®^1442 stimulates cardiomyogenesis and angiogenesis from murine and human embryonic stem cells by promoting differentiation of cardiovascular progenitors rather than proliferation. Bioassay-guided profiling points to high-molecular-weight oligomeric procyanidin-containing fractions as drivers of this activity [97]. These findings suggest a novel pharmacology for hawthorn that extends beyond symptomatic modulation to potential support of reparative processes, although translation to in vivo regeneration remains to be established.

The hawthorn literature spans chemical, cellular, and whole-organism preclinical models (in vitro radical scavenging and molecular assays [88,93]; ex vivo myocardial contractility and endothelial fractionation experiments [92,103]; in vivo cardiac injury and metabolic models [94,98,100]) and is complemented by randomized clinical trials and multicenter evaluations of standardized extracts (e.g., WS^®^1442) that attest to symptomatic and functional cardiovascular benefits in specific populations [85,96,99,103]. However, heterogeneity in species, plant parts, extraction methods, and dose regimens complicates direct generalization across studies [83,86,88].

#### 3.1.4. General Conclusion

Overall, *Crataegus monogyna* exhibits a multi-modal cardioprotective profile supported by a chemically complex phytochemistry dominated by polyphenols (notably oligomeric procyanidins and flavonoids), with additional contributions from triterpenes and other minor constituents [88,92,93,94,97]. Preclinical evidence supports effects on oxidative stress/ROS handling, inflammatory signaling, endothelial function (including NO-related vasodilation), lipid-related cardiometabolic parameters, and tolerance to ischemia–reperfusion–relevant insults; however, much of the mechanistic literature remains model-dependent [88,92,93,94,97]. In humans, studies using standardized preparations (e.g., WS^®^1442) suggest improvements mainly in symptoms and surrogate/functional outcomes such as endothelial function (FMD) and selected heart-failure related measures, whereas evidence for hard cardiovascular endpoints remains limited [85,96,99,103]. Hawthorn extracts are generally well-tolerated in clinical studies, but systematic pharmacokinetic characterization and interaction assessment in cardiovascular polypharmacy are still incomplete [81,99,105]. From a dosing perspective, the clinical literature for standardized hawthorn extract WS^®^1442 is largely anchored around ~900 mg/day, while dose-ranging exposures up to ~1800 mg/day have been evaluated with acceptable tolerability. Importantly, available dose-comparison data do not consistently show incremental benefit at the higher dose on vascular/functional surrogate endpoints, suggesting a possible plateau within commonly studied ranges and underscoring the need for PK/PD-linked dose–exposure analyses in future trials [81,88,92,93,94,97,99,105].

To strengthen translatability, priority should be given to (i) pharmacokinetic and bioavailability studies that define exposure to key constituents/metabolites and establish PK/PD relationships, including herb–drug interaction potential with common cardiovascular therapies, and (ii) harmonized clinical evaluation using standardized, chemotype-controlled formulations and pre-specified, standardized endpoint sets that clearly distinguish surrogate vascular/metabolic outcomes from hard cardiovascular endpoints in adequately powered randomized trials [81,88,97,99].

Collectively, the body of evidence positions *Crataegus* as a scientifically plausible source of cardioprotective phytopharmacology with a favorable safety profile and multiple mechanistic pathways amenable to deeper translational and clinical investigation.

### 3.2. Allium sativum *L.* (Garlic)

#### 3.2.1. Historical Background

*Allium sativum* L. (garlic, Amaryllidaceae family) has a long history as both a culinary ingredient and a medicinal plant, with widespread traditional use for circulatory and cardiometabolic complaints across European, Ayurvedic, and Traditional Chinese Medicine (TCM) practices [106,107]. These historical indications have stimulated extensive experimental and clinical investigation of garlic-derived preparations as potential cardioprotective adjuncts, particularly in relation to blood pressure and lipid-related risk factors. Importantly, the evidence base is shaped by variability among formulations (e.g., raw garlic, powders, oils, and standardized preparations such as aged garlic extract), which differ in organosulfur composition, bioavailability, and tolerability. Consequently, mechanistic findings are predominantly derived from experimental models, whereas human data are heterogeneous and largely focused on surrogate cardiometabolic endpoints rather than hard cardiovascular outcomes [106,107,108].

#### 3.2.2. Relevant Cardioprotective Phytocompounds

The cardioprotective activity of garlic is primarily attributed to organosulfur compounds (OSCs), with additional contributions from polyphenols/flavonoids, saponins, and polysaccharides that vary across bulb tissues and processing fractions [106,107,109]. In intact cloves, cysteine sulfoxides—particularly alliin (S-allyl-L-cysteine sulfoxide) and related γ-glutamyl derivatives—serve as key precursors [110,111]. Upon mechanical disruption (crushing, chopping, chewing), alliinase converts alliin to allicin (diallyl thiosulfinate), a highly reactive and unstable intermediate that rapidly decomposes into multiple oil- and water-soluble OSCs (e.g., diallyl sulfide (DAS), diallyl disulfide (DADS), diallyl trisulfide (DATS), ajoenes, and vinyldithiins) [110,112]. Aged or processed preparations are enriched in more stable, water-soluble metabolites such as S-allylcysteine (SAC) and S-allylmercaptocysteine (SAMC), which are frequently measured as bioactive markers and may better reflect in vivo exposure than allicin itself [113,114]. Beyond OSCs, garlic also contains phenolic constituents (e.g., quercetin) that can contribute to antioxidant and vasoactive effects, although these are generally considered secondary to OSC-driven activity [106,109,115].

Importantly, garlic phytochemistry is highly sensitive to cultivar/ecotype (e.g., purple vs. white cultivars), tissue composition, storage, and processing, leading to substantial differences in OSC yield and speciation across products. This variability is a major determinant of reproducibility and complicates direct comparison of mechanistic and clinical findings unless preparation methods and standardization markers are explicitly reported [110,116].

#### 3.2.3. Mechanisms of Cardioprotective Effects and Medicinal Benefits

Mechanistic and efficacy evidence for garlic spans in vitro studies, ex vivo tissue models, and in vivo animal experiments, whereas human trials remain heterogeneous and are largely focused on surrogate cardiometabolic endpoints rather than hard cardiovascular outcomes [106,107,108,109,117]. Interpretation is further shaped by formulation-dependent differences in organosulfur composition, bioavailability, and dosing (e.g., raw garlic, oils, powders, aged garlic extract), which can confound cross-study comparability [110,111,113,114]. Within this evidence hierarchy, garlic—driven predominantly by organosulfur compounds (OSCs) and supported by polyphenols and other minor constituents—has been reported to modulate antioxidant/ROS pathways, inflammatory signaling (including NF-κB and Nrf2-related programs), endothelial/vasoactive regulation (notably H_2_S and NO pathways), lipid-related and anti-atherosclerotic mechanisms, cell-death/remodeling processes, and mitochondrial function [106,107,108,109,117]. The principal mechanisms, with types of evidence, are summarized below:

- Antioxidant and ROS scavenging:

Garlic extracts and isolated OSCs exert direct radical scavenging activity and indirectly enhance endogenous antioxidant defenses across experimental systems [106,108,109,118]. Ex vivo and in vivo investigations report reductions in lipid peroxidation markers (e.g., TBARS), attenuation of oxidative damage, and induction of antioxidant responses after garlic or OSC treatment [109,112,118,119]. Mechanistically, several garlic constituents have been reported to activate Nrf2-dependent antioxidant signaling and reduce oxidative stress predominantly in cellular and animal models. For example, allicin and related compounds have been associated with Nrf2 pathway modulation and downstream antioxidant gene expression in cellular systems [109,120,121].

- Anti-inflammatory signaling (NF κB, cytokine modulation):

Multiple studies have reported that garlic OSCs can suppress pro-inflammatory signaling and cytokine production in vitro and in animal models, including inhibition of NF-κB activation and reductions in mediators such as TNF-α and interleukins [106,111,120,121]. Ex vivo models and animal experiments show attenuation of inflammatory responses after garlic extract or isolated OSC administration, and mechanistic reports implicate cross-talk between Nrf2 activation and NF κB suppression as part of the protective signature [109,118,120,121]. Clinical studies assessing inflammatory biomarkers have yielded variable results and are generally limited to surrogate measures, emphasizing that translation of anti-inflammatory mechanisms into consistent clinical benefit likely requires standardized preparations, exposure-aware dosing, and adequately powered trials [109,122,123].

- Endothelial protection and NO/H_2_S regulation:

Garlic-derived OSCs influence vascular tone and endothelial function by modulating gaseous signaling mediators: hydrogen sulfide (H_2_S) production and nitric oxide (NO) signaling. Benavides et al. reported in preclinical models that OSCs can stimulate H_2_S generation in thiol-containing systems and proposed H_2_S-mediated relaxation of vascular smooth muscle via KATP channel activation as a major vasoactive mechanism [117]. Subsequent animal studies corroborate an H_2_S-dependent component of garlic’s vasoregulatory and cardioprotective effects, and S-allylcysteine or related metabolites have been implicated in these pathways in disease models (e.g., diabetic cardiomyopathy) [108,117,119]. Ex vivo isolated heart experiments with hydroalcoholic garlic extracts further support endothelial/vascular protective effects under oxidative and ischemic stress conditions [109].

- Lipid-lowering and anti-atherosclerotic effects:

Human trials most consistently report modest effects on blood pressure and lipid parameters, with substantial between-study heterogeneity and limited endpoint standardization [111,122,123].

Clinical trials indicate that certain garlic preparations can produce modest but statistically significant reductions in total cholesterol and low-density lipoprotein (LDL) cholesterol, as well as improvements in cardiometabolic indices in some populations, while heterogeneity in study design, dose, duration, and garlic formulation leads to inconsistent findings across studies [111,122,123]. Notably, most trials evaluate lipid profiles and related cardiometabolic surrogates rather than hard cardiovascular endpoints, which remain insufficiently studied for garlic preparations. Preclinical mechanistic work implicates OSC modulation of hepatic lipid metabolism (e.g., downregulation of lipogenic regulators such as SREBP-1 and ACC) and systemic effects mediated in part via alterations of the gut microbiome that favor improved lipid handling. These pathways have been described in dietary and obesity/non-alcoholic fatty liver disease models treated with garlic oil or defined OSCs [107,111,112]. Studies highlight that the precise active hypolipidemic moiety among allicin, SAC, DATS, and other OSCs remains uncertain, complicating therapeutic standardization [123,124].

- Anti-apoptotic and anti-fibrotic pathways:

In experimental cardiac injury models, garlic preparations and OSCs have been reported to reduce apoptotic signaling and improve contractile indices. In streptozotocin-induced diabetic rats, garlic oil supplementation reduced myocardial apoptosis and improved contractile indices, a benefit linked mechanistically to H_2_S signaling and to SAC activity in vivo [119]. At the molecular level, allyl sulfur compounds modulate apoptotic regulators (Bcl 2 family proteins, caspases, p53) in cell models and in vivo tissues, consistent with an ability to stabilize cell survival pathways in stressed myocardium and to reduce maladaptive remodeling [109,113,125]. These anti-apoptotic effects are reported across isolated cell systems, rodent disease models, and ex vivo heart preparations, although clinical evidence addressing cardiac remodeling remains limited [109,119,125].

- Mitochondrial protection and energy metabolism modulation:

OSCs preserve mitochondrial integrity by maintaining membrane potential, limiting mitochondrial swelling and cytochrome C release, and attenuating mitochondrial lipid peroxidation in isolated mitochondria and tissue models [109,112,113]. Such mitochondrial stabilization can translate into improved myocardial resilience to ischemia–reperfusion injury and metabolic insults in animal and ex vivo models, providing a plausible mechanistic bridge between cellular protection and organ-level cardiac function [109,119]. The variety of OSCs (oil and water-soluble) differs in mitochondrial bioactivity and cellular uptake, and metabolic conversion (e.g., to SAMC) modulates both potency and pharmacokinetics [113,114,124].

- Antiplatelet and antithrombotic actions:

Garlic exhibits antiplatelet and anticoagulant activities in multiple preclinical and some clinical reports, which may contribute to reduced atherothrombotic risk but also carries the potential for interaction with antithrombotic drugs. Reviews of garlic’s cardiovascular effects therefore recommend consideration of platelet modulating activity when garlic is used concomitantly with anticoagulant or antiplatelet therapies and emphasize the need for clinical monitoring in those settings [107,108].

The mechanistic and efficacy evidence for garlic spans in vitro cell culture and isolated mitochondria or tissue studies [113,121,125]; in vivo rodent disease models demonstrating improvements in blood lipids, blood pressure surrogates, myocardial function, and reduced apoptosis [112,114,119]; ex vivo isolated heart protection [109]; and randomized clinical trials showing modest cardiometabolic benefit but considerable between-study heterogeneity [111,122,123]. Dose–response interpretation in humans is formulation-dependent. Across hypertension and cardiometabolic trials, the most commonly studied regimens include standardized garlic powder (~600–900 mg/day; typically characterized by allicin yield) and aged garlic extract (often ~1.2 g/day, standardized to S-allyl cysteine). A dose–response program with aged garlic extract suggests a threshold/plateau pattern (a moderate daily dose achieving BP reductions, with no clear added benefit at higher dosing), highlighting why standardized chemistry and exposure-guided dosing are essential for reproducible clinical translation. A persistent translational challenge is formulation and bioavailability: allicin is generated only after tissue disruption and is chemically labile; metabolism in the gut and reactions with dietary thiols alter circulating OSC species, and different products (raw garlic, garlic oil, aged garlic extract, powdered preparations) yield divergent OSC profiles and pharmacokinetics. These factors underlie variability in clinical outcomes and complicate dose-effect interpretation [110,111,123,124].

#### 3.2.4. General Conclusion

Overall, garlic (*Allium sativum* L.) is supported by a substantial preclinical literature and a growing, albeit heterogeneous, clinical literature as a multitarget cardioprotective botanical whose primary bioactivity is mediated by organosulfur chemistry (including allicin-derived sulfur species and the more stable water-soluble metabolites SAC and SAMC), with additional contributions from polyphenols and other constituents [106,107,110,111]. Mechanistically, garlic exerts antioxidant and anti-inflammatory effects, modulates endothelial vasoactive pathways (notably via H_2_S and NO), favorably influences lipid metabolism (including via gut microbiome interactions), stabilizes mitochondria, and reduces apoptotic signaling, actions documented in cell, isolated organ, and animal models and only partly reflected in human studies that primarily assess surrogate cardiometabolic outcomes (e.g., lipids and blood pressure), with limited evidence for hard cardiovascular endpoints [109,111,113,117,122,123]. Accordingly, translation would benefit from standardized, chemotype-controlled formulations with defined OSC markers and exposure-aware dosing, alongside adequately powered randomized trials that pre-specify harmonized endpoint sets, distinguishing surrogate vascular/metabolic and biomarker readouts (e.g., endothelial function, inflammatory markers, and NO/H_2_S-related measures) from hard cardiovascular outcomes, to clarify efficacy and dose–response relationships [108,110,116,123,124,126]. Safety considerations, including gastrointestinal tolerability, inter-individual differences in bioavailability/metabolism, and potential interactions with antithrombotic therapy, should be addressed prospectively through pharmacokinetic and herb–drug interaction studies and integrated into clinical development and practice recommendations [106,107,108]. Taken together, the preclinical mechanistic convergence and the suggestive clinical signal support continued rigorous translational research on garlic-derived compounds as candidate cardioprotective agents, with emphasis on standardized chemistry, pharmacokinetics, mechanism-directed biomarkers, and well-designed clinical trials.

### 3.3. Olea europaea *L.* (Olive)

#### 3.3.1. Historical Background

*Olea europaea* L. (olive, Oleaceae family) and its products, most notably olive oil, are central to Mediterranean dietary patterns and have longstanding use in European traditional medicine, where leaf preparations and oil consumption were historically linked to circulatory and metabolic health [127,128,129]. Contemporary research has connected olive-derived interventions with the modulation of cardiovascular risk factors such as blood lipids, blood pressure, and low-grade inflammation, providing a mechanistic rationale for cardioprotective effects. However, the strength of human evidence varies by preparation and is often based on surrogate risk markers rather than hard cardiovascular endpoints [127,128,130,131]. Importantly, olive leaves and other non-culinary fractions (e.g., pomace and olive-mill by-products) can be enriched in phenolics and are increasingly explored for functional foods and nutraceuticals, but their translational interpretation depends on the rigorous characterization and standardization of phenolic profiles across sources and processing conditions [132,133,134].

#### 3.3.2. Relevant Cardioprotective Phytocompounds

The cardioprotective bioactivity of *Olea europaea* is mainly attributed to (i) the phenolic fraction of olive-derived products—encompassing polyphenols and secoiridoids—and (ii) the monounsaturated fatty acid profile of olive oil. Across olive matrices, the most emphasized bioactive phenolic groups include secoiridoids (notably oleuropein and related ligstroside derivatives), simple phenolic alcohols (hydroxytyrosol and tyrosol), and phenolic aldehydes such as oleocanthal and oleacein, with additional contributions from flavonoids and other minor phenolics (e.g., phenolic acids, flavanols, verbascoside, and lignans) [128,131,135,136].

Compositional analyses consistently indicate compartmentation across plant parts and products: extra-virgin olive oil (EVOO) and fruits are relatively enriched in hydroxytyrosol/tyrosol and oleocanthal/oleacein, whereas olive leaves typically accumulate higher concentrations of oleuropein and related secoiridoids [135,136,137]. Critically, cultivar, processing, storage, and agronomic stressors (e.g., water deficit, salinity) can substantially shift phenolic abundance and speciation, making phenolic profiling and marker-based standardization (e.g., key secoiridoids and hydroxytyrosol-related metrics) central to reproducibility and translational interpretation [135,136,137]. Hydroxytyrosol, a principal in vivo derivative of oleuropein, is often highlighted for antioxidant and anti-inflammatory activity, while oleocanthal and oleacein have attracted interest for distinct bioactivities. In contrast, the mechanistic contribution to the overall atheroprotective signal of several minor classes (lignans, verbascoside) remains less well-defined [131,137,138,139,140].

#### 3.3.3. Mechanisms of Cardioprotective Effects and Medicinal Benefits

Mechanistic evidence for cardioprotection by olive-derived constituents is extensive but derives predominantly from chemical/cellular systems and animal models, while human evidence is largely drawn from dietary patterns and intervention studies that primarily assess intermediate (surrogate) risk markers rather than hard cardiovascular outcomes [128,130]. Interpretation is also preparation-dependent, as phenolic content and speciation vary markedly across EVOO, leaf extracts, and phenolic-enriched products depending on cultivar and processing, making phenolic profiling and standardization essential for reproducibility and translation [135,136,137]. The principal mechanistic themes include antioxidant/redox modulation, inflammatory signaling control, endothelial protection, lipid and metabolic regulation, anti-platelet/anti-atherogenic effects, and modulation of cell survival and energy metabolism.

- Antioxidant and ROS scavenging:

Hydroxytyrosol, oleuropein, and related phenols act as direct radical scavengers and chain-breaking antioxidants in chemical and cellular assays, reduce markers of oxidative damage (protein carbonyls, 4-hydroxynonenal), and limit LDL oxidation in experimental systems, thereby opposing a key early driver of atherogenesis and endothelial dysfunction [131,140,141,142]. Beyond direct scavenging, olive phenolics activate endogenous cytoprotective programmes; for example, hydroxytyrosol induces heme oxygenase 1 (HO 1) via Nrf2 stabilization in vascular endothelial cells, linking antioxidant chemistry to regulated redox signaling and improved cellular resilience [127,143,144,145].

- Anti-inflammatory signaling (NF κB, cytokine modulation, eicosanoid pathways):

Olive phenolics blunt pro-inflammatory pathways at multiple levels. Several studies show suppression of NF κB and AP 1 activation and consequent downregulation of adhesion molecules, cytokines, and matrix-remodelling enzymes in endothelial cells and immune cells [146,147,148]. For example, HT and other EVOO polyphenols attenuate TNF α-induced ROS production and NF κB signaling and reduce expression of MCP 1, CXCL10, COX 2, and MMP 2 in cell models, while oleuropein inhibits LPS-induced inflammatory responses in macrophages and zebrafish and reduces pro-inflammatory cytokine production in vitro and in vivo [146,148,149,150]. In parallel, inhibition of eicosanoid biosynthesis (lipoxygenase and downstream leukotriene formation) by oleuropein, hydroxytyrosol, and related phenols has been demonstrated in leukocyte models, providing a mechanistic basis for reduced leukocyte recruitment and vascular inflammation [149,151,152]. These anti-inflammatory actions are well-documented in experimental systems and provide biological plausibility for vascular benefit. However, direct evidence for coronary artery disease prevention or treatment in humans remains limited and requires confirmation in standardized, endpoint-driven clinical trials [149,152,153].

- Endothelial protection and nitric oxide regulation:

Olive phenolics protect endothelial cells from oxidative and inflammatory insults and suppress endothelial activation, specifically reducing expression of VCAM 1, ICAM 1, and E-selectin, thereby limiting leukocyte adhesion and early atherogenic events in vessel walls [131,146,148]. Hydroxytyrosol and its major circulating metabolites preserve endothelial function in human aortic endothelial cells and reduce markers of dysfunction in vitro. HT-dependent induction of HO-1 (Nrf2 pathway) contributes to endothelial repair and resilience, linking redox regulation to maintenance of endothelial homeostasis [127,143,148]. In inflammatory cell systems, HT also modulates nitric oxide pathways and inducible nitric oxide synthase (iNOS)-dependent responses, supporting a role for olive phenolics in balancing NO signaling under inflammatory stress [147,148].

- Lipid-lowering and anti-atherosclerotic effects:

Olive oil and leaf phenolics act additively with the monounsaturated fatty acid matrix to produce favorable effects on lipid metabolism and atherogenesis. Experimental work shows modulation of adipocyte transcriptional programmes, improvements in lipid and glucose handling (notably by oleacein and hydroxytyrosol), a reduction in LDL oxidation, and beneficial changes in plasma lipoprotein profiles in preclinical models, while population studies and diet-based interventions implicate olive oil consumption in lower cardiovascular risk [130,152,154,155]. Notably, most human studies in this area focus on lipid profiles, blood pressure, and inflammatory/oxidative biomarkers, whereas evidence for hard cardiovascular endpoints attributable to specific olive phenolics is still limited. Whole-genome transcriptomics in adipocytes exposed to oleacein identifies pathways linked to lipid and glucose metabolism and inflammation, linking a defined olive phenolic to specific metabolic gene networks [154]. Preclinical dietary studies of olive leaf extracts and phenolic-rich preparations report attenuation of cardiac, hepatic, and metabolic perturbations induced by obesogenic diets, and comparative studies indicate that both oleuropein and hydroxytyrosol-rich extracts can ameliorate high-fat diet-induced dyslipidaemia and liver injury in rats [130,152,154].

- Anti-apoptotic and anti-remodelling effects:

Isolated secoiridoids such as oleuropein exert cytoprotective and anti-apoptotic effects in tissue injury models. For example, attenuation of cisplatin-induced renal apoptosis via inhibition of ERK signaling has been reported, and olive leaf extracts reduce pathological cardiac and hepatic remodeling in diet-induced rodent models, suggesting potential for limiting maladaptive post-injury cardiac remodeling in preclinical systems [130,138,152]. While direct evidence for anti-fibrotic effects in human cardiac pathology remains limited, the preclinical data indicate plausibility for modulation of cell survival and fibrotic pathways by olive phenolics [128,152].

- Mitochondrial protection and energy metabolism modulation:

Emerging data from in vitro and transcriptomic studies indicate that olive phenolics influence energy metabolism, mitochondrial resilience, and redox-sensitive metabolic signaling, effects that are mechanistically relevant to cardiomyocyte energetics and systemic metabolic risk factors for cardiovascular disease [137,143,154]. These pleiotropic metabolic actions support the capacity of olive phenolics to act both on the vascular compartment and on metabolic tissues that drive cardiometabolic risk.

- Anti-platelet and anti-thrombotic properties:

Hydroxytyrosol, tyrosol, and other olive phenols exhibit anti-platelet and anti-PAF (platelet-activating factor) biosynthesis effects in cellular systems and have been associated with reduced platelet activation in experimental models, providing a mechanistic rationale for reduced thrombotic risk in the context of atherosclerotic disease [155,156,157].

- Epigenetic and transcriptomic modulation (nutrigenomic effects):

Beyond classical biochemical mechanisms, olive phenolics exert epigenetic and transcriptomic effects (microRNA modulation, histone modifications, and gene expression changes) that may underlie long-term modulation of inflammatory and metabolic pathways. Experimental omics studies document these epigenetic signals as a promising avenue for durable nutraceutical effects and precision nutrigenomics approaches [143,144,145,154]. These regulatory layers offer mechanistic bridges between dietary exposure, chronic disease risk modification, and individual variability in responses.

The mechanistic profile above is supported by a spectrum of data types: in vitro work using endothelial cells, macrophages, and adipocytes documents molecular targets and pathways (e.g., NF κB, AP 1, Nrf2/HO 1, 5-LOX) [146,148,150,151]; in vivo experiments (rodents, zebrafish) demonstrate organ-level benefits and improvements in lipid, inflammatory, and structural endpoints [130,138,152]; omics and transcriptomics approaches reveal pathway-level reprogramming by specific phenolics (e.g., oleacein) [154]; and human observational and dietary intervention literature (including Mediterranean-diet centered epidemiology and enriched-oil functional food studies) support associations between olive product consumption and lower cardiovascular risk, although high-quality randomized trials of isolated phytochemicals remain limited and are an active area for translation [128,130,149,156]. Pharmacokinetic studies indicate rapid metabolism of secoiridoids (oleuropein → hydroxytyrosol → conjugated metabolites) and renal excretion predominantly as glucuronides and sulfates, an important consideration for dosing, bioavailability, and clinical translation [137,138,139]. Safety and toxicology assessments of hydroxytyrosol and related phenolics are favorable in preclinical studies, and a growing body of human data supports tolerability at dietary and supplemental exposures, yet systematic clinical dose–response and long-term safety trials are still needed [128,156].

Overall, *Olea europaea* provides a spectrum of phenolic constituents (e.g., hydroxytyrosol-related species, oleuropein-derived secoiridoids, oleacein, and oleocanthal) that, predominantly in experimental models, have been reported to converge on redox-adaptive (Nrf2/HO-1) and inflammatory (NF-κB-related) pathways, preservation of endothelial homeostasis, modulation of platelet/eicosanoid signaling, and favorable lipid–glucose regulatory networks [128,131,140,146]. These mechanistic observations are broadly consistent with epidemiological and dietary-intervention literature linking olive-centered dietary patterns—particularly EVOO-rich diets—to improved cardiometabolic risk profiles and lower cardiovascular risk, but causal attribution to individual phytochemicals and demonstration of hard endpoint benefit remain areas for further translation [129,130,149].

Key research directions include adequately powered randomized trials of phenolic-enriched EVOO and chemotype-controlled standardized extracts with harmonized endpoint sets (clearly separating surrogate biomarkers from hard outcomes), PK/PD-informed dosing and exposure biomarkers, and mechanistic human studies (endothelial function, platelet reactivity, and omics-informed signatures) that account for processing/stability effects and inter-individual response variability [133,145,154].

#### 3.3.4. General Conclusion

Overall, *Olea europaea* L. provides a chemically diverse repertoire of phenolic constituents that, predominantly in experimental models, have been reported to modulate redox and inflammatory pathways, support endothelial homeostasis, and influence lipid-related and platelet-associated processes. In humans, the most consistent evidence derives from olive-centered dietary patterns and phenolic-enriched interventions that primarily improve intermediate (surrogate) cardiometabolic markers, while demonstration of hard cardiovascular endpoint benefit attributable to specific phytochemicals remains limited. These considerations justify continued translational efforts to define standardized, chemotype-controlled formulations, PK/PD-informed dosing regimens, and clinically meaningful, harmonized endpoints that can establish efficacy and safety with reproducible bioavailability [128,133,140,154,155,156].

### 3.4. Ginkgo biloba *L.*

#### 3.4.1. Historical Background

*Ginkgo biloba* L. (Ginkgoaceae family) has long-standing use in traditional Chinese medicine for conditions linked to impaired circulation and was later adopted in European phytotherapy, particularly for cerebrovascular insufficiency and peripheral circulatory symptoms such as intermittent claudication [158,159]. In modern phytopharmacology, *Ginkgo* is most closely associated with standardized leaf extracts (notably EGb 761), which provide a defined chemical profile and have been evaluated extensively in experimental models and, to a more limited and heterogeneous extent, in human studies relevant to vascular function [158,159,160,161]. These trans-cultural uses and the availability of standardized preparations have motivated continued research into *Ginkgo* leaf extracts as multi-constituent agents capable of engaging conserved pathways implicated in ischemia/reperfusion injury, endothelial dysfunction, and related cardiovascular contexts, while highlighting the need to interpret cardioprotective claims in light of evidence hierarchy and endpoint selection [158,159,161].

#### 3.4.2. Relevant Cardioprotective Phytocompounds

The cardiovascular bioactivity of *Ginkgo* leaf preparations is primarily attributed to two dominant phytochemical families: (i) flavonoid glycosides (largely quercetin-, kaempferol-, and isorhamnetin-derived flavones/flavonols) and related polyphenols, and (ii) terpene trilactones (ginkgolides A–C and bilobalide) [160,162,163]. Evidence for the predominance of these phytocompounds in standardized preparations is exemplified by EGb 761 (typical composition: ≈24% flavone glycosides, ≈6% terpenes including ginkgolides and bilobalide) and by pharmacokinetic analyses that quantify ginkgolides and bilobalide in plasma after oral dosing [160,162,164].

Additional constituents (e.g., smaller phenolic acids, proanthocyanidins, and polysaccharide fractions) contribute to redox-related and immunomodulatory effects in experimental studies, but they are generally less emphasized in standardized leaf extracts and cardiovascular translation [164,165]. In mechanistic literature, the most frequently cited cardiovascular-relevant constituents include the flavonoid fraction and terpene trilactones—particularly ginkgolide B (often discussed in relation to PAF signaling) and bilobalide (reported to influence inflammatory and cell-survival pathways)—with most evidence deriving from preclinical models [162,166,167].

#### 3.4.3. Mechanisms of Cardioprotective Effects and Medicinal Benefits

The cardioprotective profile of *Ginkgo biloba* extracts is attributed to multi-target actions of their major constituent classes (primarily flavonoids and terpene trilactones, with potential contributions from other fractions) that have been reported to influence oxidative stress, inflammation, endothelial function, platelet reactivity, mitochondrial integrity, and related signaling networks. Mechanistic support derives predominantly from in vitro and animal models (e.g., cardiac I/R, drug-induced cardiotoxicity, atherosclerosis paradigms), whereas human evidence is heterogeneous and largely focused on cerebrovascular and peripheral circulatory outcomes rather than hard cardiovascular endpoints [159,161,162,168]. The principal mechanisms, with types of evidence, are summarized below:

- Antioxidant and reactive oxygen species scavenging:

*Ginkgo* leaf flavonoids and proanthocyanidins exhibit direct free-radical scavenging and redox-modulatory activities that reduce lipid peroxidation (e.g., lower malondialdehyde) and restore endogenous antioxidant enzymes (SOD, CAT, GSH) in cell and animal models. Antioxidant synergy among flavones, proanthocyanidins, and organic acids has been demonstrated in vitro and underlies part of the extract’s protective effect against oxidative injury [163,169,170]. These antioxidant actions have been mechanistically linked to reduced oxidative stress in endothelial cells and myocardium in experimental ischemia/reperfusion and cardiotoxicity models [158,159].

- Anti-inflammatory signaling:

Preclinical studies suggest that multiple *Ginkgo* constituents can modulate inflammatory cascades. For example, polysaccharide fractions and leaf-derived constituents have been reported to attenuate NF-κB and MAPK signaling in macrophage and endothelial models and to influence cytokine-related regulatory networks (including microRNA-associated pathways) in experimental systems [165,167,171,172]. In animal and cellular models, these anti-inflammatory effects act in parallel with redox modulation to reduce endothelial activation and vascular inflammation relevant to atherogenesis [173,174].

- Endothelial protection and NO/HO-1 signaling:

*Ginkgo* preparations protect endothelial cells by attenuating oxidative stress-induced adhesion molecule expression and by activating cytoprotective enzyme systems. Notable mechanisms include the induction of heme oxygenase-1 (HO-1) and activation of PI3K/Akt → eNOS signaling in endothelial progenitor and endothelial cells, with downstream increases in NO bioavailability, improved vasorelaxation, and facilitation of vascular repair in experimental vascular injury models [173,175,176].

- Lipid-lowering and anti-atherosclerotic effects:

Preclinical studies suggest that *Ginkgo* extracts attenuate atherogenesis through combined redox, inflammatory, and metabolic actions. Proposed mechanisms include Nrf2-linked cytoprotective signaling in vascular models and, in some experimental settings, associations with altered gut microbiota composition and microbial metabolites that could influence systemic lipid and inflammatory homeostasis [174,177]. While these findings support pleiotropic anti-atherosclerotic hypotheses, translation to consistent human lipid or atherosclerosis-related endpoints remains uncertain and requires controlled trials using standardized preparations and predefined outcomes [177].

- Anti-apoptotic and mitochondrial protective pathways:

*Ginkgo* phytoconstituents prevent programmed cell death through multiple mitochondrial targets: flavonoids (e.g., quercetin, myricetin) can inhibit apoptosis-inducing factor translocation from mitochondria to nucleus, bilobalide attenuates p53- and caspase-dependent mitochondrial apoptosis signaling, and extract-level treatments up-regulate Bcl-2 and down-regulate Bax in endothelial and cardiac models, thereby preserving mitochondrial membrane potential and reducing cytochrome-C release in studies of cardiotoxicity and I/R injury [166,167,169]. These mitochondrial actions underlie reported reductions in infarct size and improved cellular viability in animal experiments but require confirmation for hard clinical cardiovascular endpoints [158].

- Antiplatelet and antithrombotic activity:

Terpenic ginkgolides (especially ginkgolide B) exert antagonism at the platelet-activating factor (PAF) receptor, and standardized extracts show antiplatelet properties in experimental assays. These antithrombotic effects contribute to improved microvascular flow in models of vascular insufficiency but also create a mechanistic basis for potential bleeding risk when combined with other antithrombotic therapies, a safety consideration emphasized in research of herbal agents used in cardiovascular contexts [178].

- Vasoactivity, vasorelaxation, and antihypertrophic signaling:

Through eNOS activation and NO-mediated signaling (and possibly cholinergic/M2 receptor-linked pathways), *Ginkgo* preparations can induce vasorelaxation and have been reported to counter-regulate hypertrophic signaling in experimental models. Such vasodilatory and antihypertrophic effects have been demonstrated primarily in in vitro and in vivo animal studies and are invoked to explain improvements in peripheral circulation observed clinically for intermittent claudication and related disorders [173,175].

- Pharmacokinetics, metabolomics, and synergistic interactions:

Recent pharmacokinetic and metabolomic studies emphasize that *Ginkgo* flavonoids and terpenoids are extensively metabolized, that bioactive metabolites can contribute to systemic effects, and that synergistic interactions among flavones, proanthocyanidins, and organic acids enhance antioxidant capacity in vitro. These data argue that standardized extracts (rather than single isolated constituents) better capture the multi-target cardiovascular biology of *Ginkgo* but also highlight the need to characterize active metabolites and dose–response relationships in humans [160,162,163].

The mechanistic actions above are supported predominantly by in vitro assays and animal models (e.g., cardiac ischemia/reperfusion, drug-induced cardiotoxicity, atherosclerosis, and vascular injury paradigms). Human data are mixed and often derive from studies targeting cerebrovascular/cognitive indications and peripheral circulatory outcomes (e.g., intermittent claudication), whereas evidence that *Ginkgo* preparations reduce major cardiovascular clinical events remains inconclusive. Larger randomized trials using standardized extracts and clearly defined cardiovascular endpoint sets are therefore required to establish clinical relevance in cardiovascular populations [159,161,168,169,170,177]. Safety-oriented pharmacology and pharmacokinetic studies additionally document bioavailability constraints, metabolite formation, and dose-dependent responses that must be accounted for in clinical translation [162,179].

#### 3.4.4. General Conclusion

*Ginkgo biloba* L. leaf preparations have been reported in preclinical research to exhibit a multi-modal cardioprotective signature, including antioxidant and anti-inflammatory activity, endothelial protection involving HO-1/eNOS/NO-related pathways, mitochondrial and anti-apoptotic effects, antiplatelet activity linked to PAF antagonism, and vasoactive actions. Emerging microbiome-associated anti-atherosclerotic hypotheses have also been proposed primarily from experimental studies. These convergent mechanisms are attributable to flavonoid glycosides, proanthocyanidins, and terpenic lactones (ginkgolides, bilobalide) acting in concert within standardized extracts [163,174,175]. The majority of mechanistic and efficacy data derive from in vitro and animal models, with human studies being comparatively limited and largely focused on cerebrovascular/cognitive indications and peripheral circulatory outcomes. Accordingly, while *Ginkgo* may represent a multi-target adjunct for vascular protection, robust cardiovascular translation requires randomized trials using standardized extracts and clearly prespecified cardiovascular endpoint sets, with explicit distinction between validated surrogate outcomes and hard clinical events [159,161,168,169,178,180].

Practical research priorities include (i) rigorous standardization of extract composition and PK/PD-informed dose-exposure relationships (building on EGb 761 as a reference preparation); (ii) integrated pharmacokinetic/metabolomic and herb–drug interaction studies to define circulating active metabolites and bleeding-risk modifiers in cardiovascular polypharmacy; and (iii) adequately powered randomized trials in cardiovascular-risk populations that use harmonized, clinically meaningful endpoint sets (validated surrogate measures and/or hard outcomes) alongside systematic safety monitoring during co-administration with antithrombotic therapies [159,160,161,162,177].

### 3.5. Leonurus cardiaca *L.* (Motherwort)

#### 3.5.1. Historical Background

*Leonurus cardiaca* L. (motherwort, Lamiaceae family) is a perennial herb widely used in European and parts of Asian traditional medicine and now naturalized across temperate regions [181,182]. In western herbal tradition, *Leonurus cardiaca* has been mainly applied for cardiac and circulatory complaints (such as palpitations and tachyarrhythmias), for nervous-cardiac symptoms (including anxiety and insomnia), and for female reproductive disorders. These indications are consistently documented in various phytotherapeutic compendia and modern research of the species’ pharmacology [183,184,185]. Preparations traditionally include infusions (teas), alcoholic tinctures, and standardized hydroalcoholic extracts of the aerial parts (leaves, stems, flowering tops). Such preparations have attracted experimental and pharmacopoeial attention, including European Pharmacopoeia-style extracts used in mechanistic studies [184,186]. Importantly, interpretation across studies is preparation-dependent (infusions vs. tinctures vs. standardized hydroalcoholic extracts), and much of the mechanistic evidence remains preclinical, underscoring the need for well-characterized formulations and endpoint-driven clinical evaluation in cardiovascular contexts. Parallel traditions in East Asia center on related *Leonurus* species (notably *L. japonicus*), which are prominent in Traditional Chinese Medicine for gynecological and circulatory disorders. Chemical and pharmacological similarities among *Leonurus* species warrant cross-species pharmacological inference, although species differences have been highlighted in chemotaxonomic and genetic studies [181,187].

#### 3.5.2. Relevant Cardioprotective Phytocompounds

Phytochemical investigations of *Leonurus cardiaca* (primarily aerial parts) indicate a chemically complex profile in which alkaloids and polyphenols (flavonoids and phenylethanoid glycosides) are most frequently emphasized, alongside iridoid glycosides and minor terpenoid/volatile constituents [182,183,184]. Among alkaloids, stachydrine is consistently reported in *L. cardiaca* material, whereas leonurine—although widely studied across *Leonurus taxa* and often discussed in relation to *L. japonicus*—has also been detected/quantified in *Leonurus* preparations and is commonly cited for vascular and myocardial activities in preclinical literature [188,189,190]. In motherwort extracts, recurrent polyphenolic markers include hyperoside, rutin/quercetin derivatives, and phenylethanoid glycosides such as verbascoside, with additional contributions from iridoid glycosides (e.g., ajugol, harpagide) and phenolic acids (e.g., chlorogenic acid) [184,191]. Volatile and other terpenoid/sterol constituents have also been described and may contribute context-dependent hemodynamic effects, although mechanistic attribution is less well defined [182,183]. Mostly, the aerial parts (leaves/flowering tops) undergo comprehensive analysis, with contemporary studies utilizing analytical techniques (HPLC/HPTLC, GC–MS) establishing marker constituents (e.g., hyperoside, verbascoside) useful for standardization and activity-guided isolation [191]. Given preparation-dependent variability, reporting these marker constituents is important for cross-study comparability and for translating pharmacological findings to defined formulations.

#### 3.5.3. Mechanisms of Cardioprotective Effects and Medicinal Benefits

*Leonurus cardiaca* preparations and isolated constituents have been investigated predominantly in in vitro, ex vivo, and in vivo experimental systems, with comparatively limited human evidence. Accordingly, the mechanistic literature should be interpreted as largely preclinical, while available clinical data are heterogeneous and mainly focused on surrogate outcomes (e.g., blood pressure and symptom measures) rather than hard cardiovascular endpoints. The principal mechanisms, with types of evidence, are summarized below:

- Antioxidant and reactive oxygen species scavenging:

In an ischemia model, *Leonuri herba* reduced oxidative stress markers, increased enzymatic antioxidant activities (superoxide dismutase, glutathione peroxidase), and lowered lipid peroxidation (malondialdehyde), with associated preservation of myocardial structure and function in rats. These findings are consistent with antioxidant and/or antioxidant-inducing actions of phenolic constituents and selected alkaloids in *Leonurus* preparations, as observed in experimental models [192]. Studies emphasize the attenuation of oxidative stress by leonurine and related alkaloids in cellular and animal models as a critical pharmacodynamic endpoint for cardiocerebrovascular protection [187,189].

- Anti-inflammatory signaling and cytokine modulation (NF-κB, MAPK):

Leonurine and *Leonurus* extracts have been reported to exert anti-inflammatory effects in experimental models. In inflammatory paradigms, leonurine attenuates NF-κB/MAPK-related signaling and reduces pro-inflammatory cytokine readouts. In doxorubicin-induced cardiotoxicity studies in rodents, aqueous aerial-part extracts of *L. cardiaca* were associated with lower circulating inflammatory markers (e.g., IL-6, C-reactive protein) alongside changes in cardiac injury biomarkers [193,194]. Collectively, these data support a predominantly preclinical anti-inflammatory axis that may contribute to reduced myocardial injury and remodeling [189,194].

- Endothelial protection and related signaling (SIRT1, NO-related pathways):

Stachydrine ameliorates high-glucose-induced endothelial cell senescence and sustains SIRT1 expression in endothelial cells, an effect that supports endothelial resilience to metabolic stress, implicating SIRT1-dependent cytoprotective pathways in *Leonurus*-mediated vascular protection [190]. Additional preclinical work links stachydrine and leonurine to reductions in endothelial ROS and improved microcirculatory parameters in ischemia models, indicating enhanced endothelial function and coronary microcirculation [192,195].

- Anti-hypertrophic, anti-remodeling, and mitochondrial/energy metabolism modulation:

Stachydrine attenuates pressure-overload and neurohormonal (norepinephrine, angiotensin II)-induced cardiomyocyte hypertrophy and modulates autophagy and ROS production in cardiac cells, suggesting a role in limiting maladaptive cardiomyocyte growth and oxidative injury [188,195]. Leonurine has been reported to reduce mitochondrial oxidative stress and apoptosis in ischemia/reperfusion and stroke models, which may underlie part of the compound’s cardioprotective effects in preclinical studies [187,189].

- Anti-apoptotic and cytoprotective effects:

*Leonurus* extracts and isolated leonurine reduce biochemical and histological indices of cardiomyocyte injury (decreased troponins, reduced myocardial enzyme release) in chemotherapy (doxorubicin)-induced cardiotoxicity models and ischemia experiments, supporting anti-apoptotic and membrane-protective actions in vivo [189,192,193].

- Electrophysiological and antiarrhythmic properties:

Extracts of *L. cardiaca* (including European Pharmacopoeia-type primary and refined extracts) exert measurable cardiac electrophysiological effects in ex vivo Langendorff heart preparations and in voltage-clamp studies of cultured cardiomyocytes. Experimental data indicate negative chronotropic and antiarrhythmic actions, with no pro-arrhythmic signals reported in these experimental preparations, backing the ethnopharmacological use of motherwort for palpitations and certain tachyarrhythmias [183,184,186]. These actions align with traditional uses for tachyarrhythmia and palpitations and with experimental cardiotonic identification studies in the genus [184,186].

- Antithrombotic/anticoagulant and antiplatelet effects:

Several studies and in vitro observations identify anticoagulant properties of *Leonurus* extracts (for instance, effects on thrombin activity and platelet aggregation), with leonurine reported to inhibit platelet aggregation in experimental systems. However, some in vitro data note complex interactions between polyphenols and microbial oxidative defenses, indicating that anticoagulant effects require careful evaluation in translational and clinical safety assessments [189,192,196].

- Metabolic and PPAR-related effects (lipid-lowering/anti-atherosclerotic potential):

Bioassay-guided screening of phenolic and iridoid constituents isolated from *Leonurus* spp. has revealed activity on PPAR subtypes (α, δ, γ) in reporter assays, suggesting a plausible molecular connection to lipid and glucose metabolism modulation and thus anti-atherosclerotic potential. These findings remain primarily preclinical but highlight mechanisms beyond redox and inflammation modulation [188,191].

The mechanistic literature includes in vitro cell culture models (endothelial cells, cardiomyocytes, receptor/ion-channel assays) and ex vivo heart preparations (Langendorff) that have defined electrophysiological and cellular actions. Multiple in vivo rodent studies demonstrate reduced ischemic injury, attenuation of drug-induced cardiotoxicity, and mitigation of hypertrophy and heart remodeling. Clinical data are limited and largely focus on symptom-oriented or surrogate outcomes (e.g., sedative effects and blood pressure measures), including a small controlled pilot study of an oil extract in hypertensive patients with concurrent anxiety and sleep disturbances [183,186,192,194,197]. Collectively, preclinical evidence supports antioxidant, anti-inflammatory, endothelial-protective, anti-remodeling, and electrophysiological activities for motherwort extracts and key constituents like leonurine and stachydrine, with human efficacy and safety evidence remaining limited and heterogeneous [183,187,189,194,197].

#### 3.5.4. General Conclusion

Overall, *Leonurus cardiaca* is a phytochemically rich medicinal herb whose aerial parts contain alkaloids (e.g., stachydrine and leonurine-related alkaloids), polyphenols (including flavonoids and phenylethanoid glycosides), and iridoids that, in experimental systems, have been reported to support a multi-target cardioprotective signature [182,183,184,191]. Across cellular, ex vivo, and animal models, extracts and selected constituents have been associated with redox and inflammatory modulation (including NF-κB/MAPK-related pathways), endothelial stress resilience (e.g., SIRT1-linked responses), effects on hypertrophy/apoptosis-related signaling, and measurable electrophysiological actions. However, human evidence remains limited and largely focused on symptom-oriented or surrogate outcomes [186,187,189,190,192,193,194,195,197]. Safety considerations include sedative and hypotensive effects reported in clinical/pharmacological sources, potential anticoagulant/antiplatelet activity with interaction risk in polypharmacy settings, and the need for more robust human safety characterization [183,186,196,198]. Although standardized acute, sub-acute, and chronic rodent toxicity studies are available and can inform translational dose selection, comprehensive clinical safety and dose–response data remain sparse [183,186,196,198].

Future research priorities should emphasize (i) rigorous marker-guided standardization of extracts (e.g., hyperoside/verbascoside and alkaloid-related markers) to improve reproducibility; (ii) pharmacokinetic and PK/PD-informed dosing studies, including systematic herb–drug interaction assessment with anticoagulants and antiarrhythmics; and (iii) adequately powered randomized trials in well-defined cardiovascular populations using harmonized endpoint sets that clearly distinguish validated surrogate measures from hard clinical outcomes [184,186,189,191,194,198]. In conclusion, *Leonurus cardiaca* presents a scientifically plausible, multi-modal cardioprotective profile grounded in well-documented phytochemistry and supported by convergent preclinical data, but its therapeutic integration into modern cardiovascular practice will require standardized preparations, mechanistic clarification, and appropriately powered clinical trials [182,183,191,197].

### 3.6. Melissa officinalis *L.* (Lemon Balm)

#### 3.6.1. Historical Background

*Melissa officinalis* L. (lemon balm, Lamiaceae family) is an aromatic perennial herb long cultivated across Europe and the Mediterranean and widely documented in ethnobotanical and phytotherapeutic sources [199,200]. Traditionally, it has been used for nervous-system complaints (e.g., anxiety and sleep disturbances) and gastrointestinal symptoms, with additional historical use for palpitations and functional cardiac complaints in several medical traditions [200,201]. Ethnopharmacological records and contemporary studies indicate that *M. officinalis* preparations, primarily aqueous infusions and essential oils, were traditionally used to alleviate symptoms such as palpitations, reinforcing its role as a cardiovascular tonic in historical texts [202,203]. Iranian and Persian materia medica sources enumerate a variety of cardiocirculatory uses, including for arrhythmia and heart weakness, reflecting historical therapeutic attributions that inform modern cardiovascular research on the species [201]. Today, lemon balm is utilized as an herbal tea and incorporated into nutraceutical and essential oil preparations, marking its relevance in contemporary markets and research [203,204]. Importantly, the type of preparation (aqueous infusions, hydroalcoholic extracts, and essential oils) strongly influences the chemical composition and biological effects, and the cardiovascular evidence base remains predominantly preclinical, with limited, heterogeneous human data largely centered on symptom-oriented or surrogate outcomes.

#### 3.6.2. Relevant Cardioprotective Phytocompounds

Phytochemical characterization of *Melissa officinalis* has identified key compound classes relevant to cardiovascular pharmacology: the active components are concentrated in the above-ground parts (leaves > stems), with commonly studied forms including aqueous decoctions/infusions, ethanolic extracts, and essential oils from leaves [205,206].

Across preparations, polyphenols are the most consistently reported bioactive fraction, with rosmarinic acid and related hydroxycinnamic acids (e.g., caffeic acid derivatives) frequently highlighted as dominant antioxidant constituents of leaf extracts [205,207]. Flavonoids (including quercetin- and luteolin-derived glycosides) are recurrent secondary polyphenols, while non-polar fractions can contain triterpenoid acids (e.g., ursolic and oleanolic acids) that are often discussed in relation to cardiometabolic regulation [208]. In contrast, essential oils are characterized by volatile terpenoids—typically citral (geranial/neral) alongside other constituents (e.g., citronellal, β-caryophyllene)—but show substantial genotype- and extraction-dependent variability [207]. HPLC and phytochemical surveys demonstrate that *M. officinalis* aerial extracts consist of various rosmarinic acid and phenolic compounds, multiple flavonoid aglycones and glycosides, and a genotype-dependent volatile fraction, information that supports their antioxidant, metabolic, and vasoactive activities [205,206]. Because composition differs across infusions, hydroalcoholic extracts, and essential oils, marker-based profiling (e.g., rosmarinic acid content and defined volatile chemotypes) is important for reproducibility and for interpreting mechanistic versus clinical findings.

#### 3.6.3. Mechanisms of Cardioprotective Effects and Medicinal Benefits

The principal mechanisms of cardioprotective effects and medicinal benefits, with types of evidence, are summarized below:

- Antioxidant/ROS scavenging:

A principal mechanistic axis supported by chemical analyses and bioassays is the antioxidant activity associated with phenolic acids and flavonoids. Quantitative assays (e.g., DPPH radical scavenging tests) underscore the robust antioxidant capacity of extracts from the aerial parts and flavonoid-rich fractions [205,206]. In endothelial cell systems, extracts of *M. officinalis* have been reported to mitigate oxidative damage. For instance, Safaeian et al. reported that these extracts protect human umbilical vein endothelial cells (HUVECs) against H_2_O_2_-induced oxidative stress, preserving cell viability [209]. In vivo, antioxidant-associated cardioprotection has been reported in experimental models, including a study where ethanolic leaf extracts reduced biochemical and histological signs of cardiac injury induced by bleomycin in rats, linked to their phenolic content [205,210]. Collectively, these findings align chemical antioxidant potential with reductions in oxidative endothelial and myocardial injury in preclinical studies [206,210].

- Endothelial protection and indirect vasomotor effects:

Endothelial resilience is a plausible pathway for cardioprotection, as indicated by the protective effects in endothelial cell assays [206,209]. A double-blind crossover study (surrogate outcome) reported reductions in systolic and diastolic blood pressure following lemon balm intake compared with placebo [211]. While this finding is consistent with a vasomotor/endothelial component, direct mechanistic evidence for eNOS activation after *M. officinalis* treatment remains limited. While direct evidence of endothelial NO synthase (eNOS) activation subsequent to lemon balm treatment is limited, in vitro endothelial protection data and clinical blood pressure improvements indicate an endothelial-mediated aspect to the cardiovascular effects of the herb, highlighting the necessity for further mechanistic studies focused on NO/eNOS pathways [209,211].

- Anti-inflammatory signaling:

Network pharmacology and experimental validations have indicated the modulation of inflammatory pathways as part of *M. officinalis*’ cardiometabolic actions. Computational analyses have predicted interactions of key constituents (quercetin, luteolin, ursolic/oleanolic acids, isoquercitrin) with inflammation and insulin signaling proteins such as AKT1 and PPARα, suggesting pathway enrichment in conditions like obesity and diabetes [208]. Experimental evaluations have shown that *M. officinalis*, particularly in combination with corn silk, mitigates high-fat diet-induced obesity, accompanied by a reduction in related inflammatory and metabolic perturbations in animal models, further supporting claims of anti-inflammatory benefits [212]. These combined insights propose that anti-inflammatory signaling contributes to cardiometabolic benefits, although direct molecular validation remains incomplete, necessitating further research [208,212].

- Lipid-lowering and anti-atherogenic effects:

*M. officinalis* has demonstrated tangible effects on circulating lipid levels in both clinical and preclinical investigations. A randomized controlled trial showed significant reductions in total cholesterol, triglycerides, and low-density lipoprotein levels due to lemon balm consumption, while no notable effects on high-density lipoprotein levels were observed [213]. Clinical investigations regarding patients with type 2 diabetes have corroborated these findings, revealing favorable shifts in metabolic markers alongside improvements in lipid profiles [214]. These findings suggest that combined effects on PPAR signaling, antioxidant/inflammatory modulation, and lipid metabolism could underlie the cardiovascular benefits associated with *M. officinalis*, supported by insights from pharmacological analyses of its constituents [208,213].

- Electrophysiological and anti-arrhythmic activities:

Preclinical studies examining cardiac electrophysiology highlight that extracts of *M. officinalis* can affect myocardial electrical characteristics and diminish arrhythmic incidents following ischemia–reperfusion injury [215,216]. Joukar et al. reported fewer reperfusion-induced ventricular fibrillation events in treated groups, with effects discussed in relation to amiodarone in the same experimental setting; ECG interval changes varied across groups [216]. This body of research provides preclinical evidence that *M. officinalis* may enhance cardiac electrophysiological stability during acute ischemic stress, although the detailed ionic targets remain to be clarified, potentially involving indirect antioxidative and autonomic pathways [215,216].

- Antiapoptotic/cardiotoxicity mitigation:

Several in vivo investigations employing chemotherapeutic cardiac injury models have reported cardioprotective effects related to *M. officinalis* extracts, likely mediated by antioxidant and cytoprotective actions. For example, ethanolic extracts have been shown to mitigate bleomycin-induced cardiotoxicity in rodents, reflected by improved histological and biochemical indicators of cardiac damage. Such results support the notion that *M. officinalis* could play an adjunctive role in alleviating cardiomyocyte damage under toxic stress conditions in preclinical settings [210].

- Mitochondrial and energy metabolism modulation:

Direct experimental evidence for mitochondrial protection (e.g., preservation of mitochondrial membrane potential and oxidative phosphorylation efficiency) after *M. officinalis* treatment is sparse. However, predictions from network pharmacology suggest that AKT1 and PPARα may be key targets for its major constituents. Furthermore, enhancements observed in metabolic models and clinical metabolic parameters align with energy metabolism and lipid handling modulation [208,212,214]. These convergent signals underscore the need for focused studies examining mitochondrial function and substrate oxidation following standardized lemon balm interventions [208,212,214].

- Central anxiolytic/hypnotic effects as indirect cardioprotection:

*M. officinalis* is well-established for its anxiolytic and sedative properties in both traditional and contemporary contexts. Modern literature emphasizes its applications in reducing anxiety and insomnia, with studies documenting effective routes of administration for its volatile constituents [200,217]. Given that psychological stress and autonomic dysregulation contribute to cardiovascular risk, the herb’s central effects, aiding in sympathetic tone reduction and anxiety alleviation, may provide an additional indirect pathway for cardioprotection, warranting deeper mechanistic evaluations in controlled trials [217,218].

Overall, Melissa officinalis has been investigated as a multi-constituent botanical in which rosmarinic acid-rich polyphenols, flavonoids, and triterpenoid acids are most often discussed in relation to antioxidant, anti-inflammatory, and cardiometabolic effects [205,207,208,209]. Evidence spans in vitro endothelial/antioxidant assays and animal models of chemically induced cardiotoxicity or ischemia–reperfusion stress, alongside a limited number of human studies reporting changes in surrogate outcomes such as blood pressure and lipid profiles [206,209,210,211,212,213,214,215]. While these findings support biological plausibility, clinical evidence remains heterogeneous and endpoint-limited, and interpretation depends on preparation type and standardization. A recently published study supports the presence of clinically important lipid-lowering activities (targeting total cholesterol, triglycerides, and low-density lipoproteins), whereas individual clinical trials affirm benefits in blood pressure and metabolic contexts, suggesting that *M. officinalis* might serve as a promising adjunct in addressing cardiometabolic risk when standardized formulations are appropriately applied [211,213,214].

Available data suggest a generally favorable tolerability profile for commonly used preparations (e.g., teas and standardized extracts), but systematic dose–response, long-term safety, and herb–drug interaction data in cardiovascular polypharmacy remain limited [206,213,217]. Given variability across strains and preparation methods (infusions vs. extracts vs. essential oils), marker-based standardization (e.g., rosmarinic acid content and volatile chemotype definition) is important for reproducibility and safety-consistent use [203,207].

Future research should prioritize PK/PD-informed dosing and exposure biomarkers, rigorous interaction assessment with commonly used cardiovascular medications, and adequately powered randomized trials using harmonized endpoint sets that clearly distinguish validated surrogate measures (e.g., blood pressure, lipids, endothelial function) from hard cardiovascular outcomes [201,213]. Mechanistic studies should focus on targeted validation of hypothesis-generating network predictions (e.g., PPARα/AKT1-linked pathways) and on defining relevant vascular/electrophysiological targets under standardized intervention conditions [208,213].

#### 3.6.4. General Conclusion

Overall, available evidence suggests that *Melissa officinalis* may offer cardiometabolic support, with predominantly preclinical data indicating redox/inflammatory modulation, endothelial stress resilience, and possible electrophysiological effects under experimental conditions. Human studies remain limited and largely report changes in surrogate outcomes (e.g., blood pressure, lipid parameters), and evidence for hard cardiovascular endpoints is currently insufficient. Therefore, future progress will require standardized, well-characterized preparations, PK/PD-informed dosing and exposure biomarkers, and adequately powered randomized trials with harmonized endpoint sets and targeted mechanistic validation of the most plausible molecular pathways [205,208,209,213,215].

### 3.7. Viscum album *L.* (Mistletoe)

#### 3.7.1. Historical Background

*Viscum album* L. (European mistletoe, Santalaceae family) is a semiparasitic evergreen shrub described in European materia medica for sedative/antispasmodic uses and for circulatory complaints, including ethnomedical applications related to elevated blood pressure [219,220,221]. Since the early 20th century, *V. album* preparations have been developed primarily within complementary oncology, where standardized aqueous or fermented extracts are administered (most commonly subcutaneously) and evaluated in randomized studies and safety-focused clinical programs, largely for quality-of-life and adjunctive outcomes [222,223,224,225]. Ethnopharmacological surveys and clinical reports further document the folk use of mistletoe in various regions for hypertension and related circulatory disorders, supporting its historical cardiovascular applications in traditional medicine [226,227].

#### 3.7.2. Relevant Cardioprotective Phytocompounds

Principal bioactive classes reported in *Viscum album* preparations include proteinaceous constituents (notably mistletoe lectins/viscumin and related proteins) and small-molecule fractions such as viscotoxins, saponin-glycosides (e.g., viscumneosides), polysaccharides, flavonoids, and triterpene acids, among other constituents [228,229,230]. Mistletoe lectins and viscumin have been biochemically characterized and are generally associated with immunomodulatory and dose-dependent cytotoxic activities [228,229,231]. Viscotoxins have been characterized as small, cysteine-rich thionins exhibiting membrane-interacting and cell-active properties [229,230]. Glycosidic constituents like viscumneoside III and V have been identified in *V. album* extracts and linked to modulation of chemokine expression, specifically suppression of monocyte chemoattractant protein-1 (MCP-1) [230,232]. Low-molecular-weight constituents (polysaccharides, flavonoids, triterpenes) are consistently reported in compositional surveys and are believed to contribute to the adaptogenic and immunomodulatory effects of extracts used in preclinical and clinical preparations, although the pharmacological attribution to individual small molecules remains under investigation and is highly dependent on extraction methods [233,234]. Thus, clinically and experimentally used preparations (aqueous, fermented, or proprietary extract formulations) contain a complex blend of lectins, viscotoxins, and non-peptidic constituents, which is crucial for understanding biological effects and safety [222,224].

#### 3.7.3. Mechanisms of Cardioprotective Effects and Medicinal Benefits

The mechanistic literature for *Viscum album* spans biochemical and cell-based studies, in vivo animal models, and human clinical investigations. However, contemporary research output has been disproportionately driven by the use of *Viscum album* in integrative oncology and immunology, such that cardiovascular-focused mechanistic studies are less numerous than the oncology-oriented literature [228,229,235]. This imbalance in research emphasis should not be interpreted as a lack of cardiovascular relevance because *V. album* has a well-established tradition of use for circulatory complaints, particularly blood-pressure regulation, and pertinent evidence base (including vascular/NO-related mechanisms and human blood-pressure observations) [219,220,221,226,232]. The main pathways implicated in cardiovascular-relevant mechanisms include (a) modulation of nitric oxide pathways and blood-pressure regulation, (b) immunomodulation and cytokine/chemokine regulation with potential anti-inflammatory consequences, (c) anti-angiogenic and growth-inhibitory signaling, and (d) direct cytotoxic and membrane-interactive actions of lectins/RIPs and viscotoxins that can induce cell death and immune activation. Each of these pathways has supporting literature of varying strength [226,228,229,235]. The principal mechanisms, with types of evidence, are summarized below:

- Nitric oxide pathway and blood-pressure regulation:

Human cardiovascular-relevant evidence is limited and is largely restricted to surrogate outcomes. One controlled evaluation of a *V. album* mother tincture reported reductions in systolic and diastolic blood pressure, with a proposed involvement of nitric oxide synthase-related pathways. However, mechanistic attribution in humans remains indirect and requires confirmation in well-controlled studies using defined preparations [226]. This observation provides a rationale for further investigations in endothelial cell and vascular ring models to evaluate vasorelaxation and NO bioavailability after exposure to defined *V. album* fractions [232].

- Immunomodulation and cytokine/chemokine regulation:

Multiple preclinical and clinical studies document the immune effects of *V. album* lectins and polysaccharide fractions, including stimulation and modulation of immune cell populations as well as alteration of circulating cytokines and mediators of angiogenesis in patients and animal models [231,235,236]. With respect to chemokine signaling, viscumneoside III and V have been reported to reduce MCP-1-related readouts in experimental systems, suggesting that specific constituents may modulate chemokine-linked inflammatory pathways [230,235]. However, most of the reported immunologic effects arise from oncology contexts. Direct evidence demonstrating that these effects lead to measurable cardiovascular protection or plaque stabilization is yet to be established [225].

- Anti-angiogenic and growth-inhibitory activity:

In oncology-focused preclinical and clinical studies, *V. album* extracts modulated angiogenesis mediators and inhibited tumor proliferation in xenograft models, demonstrating destabilization of oncogenic drivers (e.g., c-Myc) in animal studies. While these anti-angiogenic programs are well described in oncology contexts, their relevance to cardiovascular disease biology remains speculative and has not been rigorously evaluated in dedicated cardiovascular models [235].

- Direct cytotoxicity and membrane action mediated by lectins and viscotoxins:

At the molecular level, viscumin inhibits mammalian protein synthesis by depurination of 28S rRNA, leading to cytotoxic effects. Viscotoxins interact with and disturb cell membranes, exhibiting specific membrane activity signatures [228,229,231]. These biochemical actions elucidate the tumor-directed cytotoxic and immune engagement effects of mistletoe preparations documented in vitro and in vivo [228,230]. The presence of such potent cytotoxic proteins raises safety concerns for systemic or high-dose exposure, necessitating careful formulation and dosing in human applications [228,229].

- Adaptogenic/systemic resilience effects:

Polysaccharide-rich fractions and some low-molecular-weight constituents of *V. album* have been associated in animal studies with improvements in physical performance and characteristics indicated to have adaptogenic potential, a profile which could hypothetically influence cardiovascular risk factors through systemic metabolic and immune modulation, although these remain hypotheses that require specific cardiovascular testing [233].

In vitro mechanistic studies have demonstrated lectin/RIP activity and viscotoxin membrane interactions [228,229]; cellular immunostimulatory and cytotoxic effects of purified lectins have been reported [231]; preclinical in vivo studies (rodents, xenografts) show immunomodulation, anti-angiogenic, and growth-inhibitory activity [235,236]; and clinical evidence primarily exists from oncology trials addressing safety and quality-of-life endpoints, along with limited clinical antihypertensive evaluations [222,223,225,226]. Conversely, dedicated mechanistic studies investigating classical cardioprotective pathways, such as mitochondrial protection, direct anti-apoptotic signaling in cardiomyocytes, lipid-lowering, or anti-remodeling actions, are currently limited in the *V. album* literature, underscoring the need for targeted cardiovascular preclinical and clinical studies alongside phytochemical standardization [225,232].

*Viscum album* L. contains a range of biologically active proteins (lectins, viscumin; viscotoxins) and non-peptidic constituents (polysaccharides, flavonoids, triterpenoids, viscumneosides) that together produce immunomodulatory, cytokine-modifying, anti-angiogenic, and direct cytotoxic effects well-documented in preclinical studies and clinical oncology programs. Preliminary clinical evidence suggests an antihypertensive effect potentially mediated by nitric oxide synthase pathways [222,225,226,228,229,231].

Clinical experience with standardized extracts suggests tolerability under defined routes and dosing regimens, but safety is strongly preparation- and administration-dependent. The presence of highly bioactive proteins (e.g., viscumin) and membrane-active viscotoxins necessitates strict dose control, route-of-administration specification, and pharmacovigilance, and limits extrapolation across products [222,223,237]. In addition, host-tree effects and extraction/fermentation methods can shift composition, and clinical reports remain methodologically heterogeneous [225].

Priorities for cardiovascular research should include (i) robust product standardization with quantitative characterization of lectins, viscotoxins, and key small-molecule fractions in clinically used preparations to enable reproducible interpretation and dosing [222,237]; (ii) targeted preclinical cardiovascular mechanistic inquiries (endothelial function assays, vascular reactivity/NO studies, ischemia–reperfusion models, cardiomyocyte mitochondrial and apoptosis assays) to determine whether the immune, anti-angiogenic, and NO-modulating activities of *V. album* translate into genuine myocardial or vascular protection [226,228,229,235]; and (iii) proof-of-concept clinical trials in hypertension and thoughtfully designed cardio-oncology studies to evaluate if *V. album* preparations can mitigate systemic or cardiac toxicity related to chemotherapy, motivated by reports of reduced chemotherapy-related toxicity with mistletoe adjuncts and the known clinical burden of cardiotoxicity from cancer treatments [225]. Such a research program should incorporate standardized products, rational dose-finding, mechanistic biomarkers (NO metabolites, cytokine panels, endothelial function), and stringent safety monitoring, given the inherent biological potency of lectins and viscotoxins [228,232].

#### 3.7.4. General Conclusion

Overall, *Viscum album* is a phytochemically complex medicinal plant whose lectin/viscumin- and viscotoxin-containing preparations, together with low-molecular-weight fractions, have been reported to influence pathways relevant to vascular biology (e.g., NO-related signaling) and immunomodulation (cytokine/chemokine regulation), largely in experimental systems. However, direct evidence supporting classical cardioprotection (e.g., myocardial salvage, anti-remodeling effects, mitochondrial protection, or lipid-lowering efficacy) remains limited. Accordingly, targeted cardiovascular research, including standardized, well-characterized products, mechanistic biomarker integration, and endpoint-driven clinical trials, is required before *V. album* can be considered for routine cardioprotective use in cardiovascular care [226,228,230,231,235].

### 3.8. Camellia sinensis (*L.*) Kuntze (Green Tea)

#### 3.8.1. Historical Background

*Camellia sinensis* (green tea, Theaceae family) is one of the world’s most widely consumed beverages and has been embedded for centuries within East Asian dietary and health traditions. Modern scientific interest in green tea follows from this long cultural use and from epidemiological observations linking habitual tea consumption with lower incidence of metabolic and cardiovascular disorders [238,239]. Contemporary studies and clinical overviews therefore treat green tea both as a traditional health-promoting agent and as a subject for rigorous evaluation in cardiometabolic research, motivating numerous in vitro, animal, and human studies of its cardiovascular effects [238,240]. Importantly, cardiovascular interpretation is preparation- and dose-dependent (brewed beverage vs. concentrated extracts/capsules, catechin profile, and caffeine content), and human evidence often relies on surrogate cardiometabolic endpoints rather than hard cardiovascular outcomes.

#### 3.8.2. Relevant Cardioprotective Phytocompounds

The principal cardioprotective fraction of green tea consists of green tea polyphenols (GTPs), principally flavan-3-ols (catechins) concentrated in the leaf material used for infusion and in concentrated leaf extracts. Catechins constitute a substantial fraction of extractable solids from dried green tea leaves (often reported in the ~30–40% range), although this proportion varies with cultivar, processing, and extraction/brewing conditions [240,241].

The principal individual catechins implicated in cardiovascular bioactivity are (−)-epigallocatechin-3-gallate (EGCG; the most abundant and pharmacologically active catechin), epigallocatechin (EGC), epicatechin (EC), and epicatechin-3-gallate (ECG) [240,241,242].

Caffeine co-occurs with catechins in leaf preparations and contributes to thermogenic/metabolic effects in combination with catechins [243]. Methylated catechins (e.g., O-methylated forms found in benifuuki cultivars) and other minor phenolics can also have distinct bioactivities [244], while oxidized polyphenols (theaflavins) are characteristic of black tea rather than green tea and thus represent a different phytochemical profile [238].

The biologically active material is concentrated in the aerial leaf tissue (dried leaves and bud) and is delivered either as brewed tea (infusion) or as standardized/enriched leaf extracts and phytosome complexes used in clinical formulations (e.g., Greenselect Phytosome) [241,243]. Because brewed tea and concentrated extracts can yield markedly different catechin exposures (and safety profiles), reporting standardized catechin composition (often EGCG as a key marker) and dosing is essential for cross-study comparability and clinical translation [241,243].

#### 3.8.3. Mechanisms of Cardioprotective Effects and Medicinal Benefits

A broad mechanistic literature suggests that green tea constituents can engage pathways relevant to cardiometabolic risk modulation, including redox signaling, inflammatory and leukocyte-recruitment processes, endothelial NO-related regulation, lipid metabolism, and vascular remodeling-related signaling. Evidence strength varies by preparation (brewed beverage vs. concentrated extracts), dose, and endpoint, with many mechanistic claims supported primarily by in vitro/animal studies and human data often focused on surrogate risk markers [240,241,245,246,247]. The principal mechanisms, with types of evidence, are summarized below:

- Antioxidant and ROS-scavenging activity:

EGCG and related catechins exhibit strong in vitro free-radical scavenging activity and can act synergistically with lipid-soluble antioxidants (e.g., α-tocopherol) in reducing LDL oxidation [241,248]. Human intervention studies have reported changes in indices of total antioxidant capacity and oxidative stress biomarkers after green tea/catechin interventions in selected populations (e.g., obesity), although results vary with dose, duration, and formulation [239,249].

- Anti-inflammatory signaling and immune modulation:

EGCG has been reported to inhibit neutrophil chemotaxis in experimental systems in vitro and in vivo [248]. Methylated catechins (e.g., found in benifuuki) can reduce mast cell activation and allergic responses in animal models [244]. In humans, randomized trials report variable outcomes for inflammatory biomarkers; some have demonstrated reductions in acute-phase proteins, while others report minimal changes, indicating context-dependent anti-inflammatory effects [250].

- Endothelial protection and NO regulation:

Green tea polyphenols have been found to modulate endothelial signaling, down-regulating caveolin-1 through ERK1/2 and p38 MAPK pathways in endothelial cells, which can modify eNOS function and NO bioavailability [241]. While green tea preparations may increase NO production in cellular assays, in vivo studies show inconsistent effects on circulating NO indices in healthy human subjects, highlighting differences between cell models and systemic physiology [251].

- Lipid-lowering and anti-atherosclerotic effects:

Epidemiological studies report associations between habitual green tea consumption and more favorable lipid profiles and cardiometabolic risk [252,253]. Randomized controlled trials and meta-analyses generally indicate small-to-moderate reductions in total cholesterol and LDL cholesterol, with effect sizes varying by baseline metabolic status, dose, and extract standardization; these outcomes should be interpreted as surrogate risk-marker changes rather than hard cardiovascular endpoints [240,252]. Formulations such as Greenselect Phytosome have been evaluated in lifestyle-intervention settings and reported improvements in lipid markers in individuals with borderline metabolic syndrome [254,255].

- Anti-proliferative, anti-remodeling, and anti-apoptotic pathways:

EGCG selectively inhibits PDGF-BB-induced intracellular signaling in VSMCs and reduces proliferation and phenotypic transformation [246]. In patients with diabetic nephropathy, green tea polyphenols have been shown to lower albuminuria, with mechanisms potentially involving reduced podocyte apoptosis and decreased activation of WNT pathway mediators [247]. Additionally, EGCG regulates calcium handling and contractility in cardiomyocytes, indicating direct effects on myocardial function [245].

- Mitochondrial function and energy-metabolism modulation:

Preclinical studies show that EGCG can reduce intestinal energy absorption while increasing fat oxidation and thermogenesis in mice, contributing to reduced diet-induced weight gain [243]. Clinical studies indicate variable benefits for weight loss and metabolic parameters in humans consuming catechin-rich preparations [254,255]. Although mitochondrial pathways are implicated, direct evidence for myocardial mitochondrial protection from green tea catechins remains less well characterized [243].

- Gut microbiome and indirect metabolic effects:

Green tea polyphenols may influence gut microbiota composition and function, with preliminary evidence suggesting prebiotic-like effects in both animal and limited human studies. These changes may be one mechanism by which green tea impacts lipid metabolism and cardiometabolic risk [256,257].

Randomized controlled trials indicate modest improvements in cardiovascular risk markers following green tea consumption, particularly in metabolically compromised individuals. However, the effect size is generally modest and varies across studies, with a call for more rigorous long-term cardiovascular outcome trials [240,247,250]. Mechanistic data support cardioprotective action but require further clinical validation [241,245,246].

Documented cases of acute liver injury and, in rare instances, acute liver failure have been associated with concentrated green tea extracts and supplements, such as certain “fat burner” products, while traditionally brewed green tea has less frequently been implicated. Variability in product composition complicates causal attribution [258,259,260,261,262]. Toxicity studies in rodents indicate dose-dependent and sex-dependent hepatic effects of EGCG and its extracts, raising challenges for translating these findings to human risk assessments [263]. Some formulations, such as fermented green tea, show high-dose tolerability in animal studies, which suggests preparation type may influence toxicity [264].

Randomized trials generally find acceptable tolerability for brewed green tea and many standardized formulations, but also document adverse events, including rare hepatotoxicity. Caution and liver monitoring are advised when using high-dose extracts or multi-ingredient supplements [265]. Green tea constituents can influence drug transporters and enzymes, affecting drug metabolism, making it vital to carefully consider concomitant therapies in cardiovascular patients on multiple medications [266].

#### 3.8.4. General Conclusion

*Camellia sinensis* (green tea) provides a catechin-rich polyphenolic fraction (notably EGCG) alongside caffeine and other constituents that, across experimental systems and human intervention studies, have been associated with antioxidant/redox effects, inflammatory modulation, endothelial signaling changes, modest lipid-profile improvements, and broader cardiometabolic risk-marker shifts [241,246,248]. Human evidence most consistently supports changes in surrogate outcomes, whereas robust demonstration of reduced major cardiovascular clinical events remains limited and warrants longer-term, endpoint-driven trials [238,239,240].

From a translational perspective, brewed green tea is generally consumed as part of dietary patterns and is typically well-tolerated, while concentrated extracts can yield substantially higher catechin exposures and therefore require careful attention to product standardization, dose, and safety, particularly given reported hepatotoxicity signals in some supplement contexts [258,259,260,261]. Future research should prioritize standardized trials with harmonized endpoint sets (clearly separating validated surrogate biomarkers from hard outcomes), mechanistic human studies at achievable systemic concentrations, PK/PD-informed dosing and exposure biomarkers, and systematic evaluation of extract safety and herb–drug interaction potential in cardiometabolic polypharmacy settings [240,245,260,263].

### 3.9. Curcuma longa *L.* (Turmeric)

#### 3.9.1. Historical Background

*Curcuma longa* L. (turmeric, Zingiberaceae family) is a perennial rhizome traditionally used in culinary and medicinal systems across South Asia and beyond. Dried rhizome and its preparations are used in Ayurveda, traditional Chinese medicine, and folk phytotherapy for a variety of indications, including digestive, hepatic, inflammatory, and circulatory disorders [267,268,269]. Traditional sources and contemporary research describe turmeric use for inflammatory and circulation-related complaints, which has motivated mechanistic studies and clinical exploration of potential cardiometabolic effects [267,270]. However, interpretation across studies is strongly formulation-dependent, given major differences in curcuminoid exposure and bioavailability across turmeric powders, extracts, and enhanced-delivery preparations [267,270]. Accordingly, turmeric has become a focus for systematic pharmacological evaluation and marker-guided phytochemical standardization, with particular emphasis on defining curcuminoid content and clinically relevant exposure [268,271].

#### 3.9.2. Relevant Cardioprotective Phytocompounds

The primary cardioprotective phytochemicals in *C. longa*, mainly sourced from the rhizome, include two chemical groups—curcuminoids (diarylheptanoids) and volatile/essential oil constituents (terpenoids)—along with various phenolics, polysaccharides, and minor alkaloids [268,271,272]. Curcuminoids are predominantly curcumin, followed by demethoxycurcumin and bis-demethoxycurcumin, reported at proportions of approximately 77%, 17%, and 3%, respectively, with cyclocurcumin as a minor constituent [268,270]. The volatile fraction of the rhizome encompasses turmerones and numerous other terpenes, contributing to biological activities and potentially acting synergistically with curcuminoids. Overall, more than 200 compounds have been identified in turmeric rhizomes [268,272]. For cardioprotective pharmacology, curcumin has been the major focus of mechanistic and interventional studies, while essential oil constituents and other phenolics are explored as complementary modulators of redox, inflammatory, and vascular pathways [268,272,273]. Because systemic exposure to curcumin varies markedly across powders, extracts, and enhanced-delivery formulations, reporting curcuminoid content and formulation characteristics is essential for cross-study comparability and clinical translation [268,271,273].

#### 3.9.3. Mechanisms of Cardioprotective Effects and Medicinal Benefits

*Curcuma*-derived constituents, most prominently curcumin, have been reported to engage multiple pathways relevant to cardiovascular biology, including redox/inflammatory signaling (often discussed in relation to NF-κB), endothelial and NO-related regulation, lipid metabolism, platelet/coagulation-related processes, and mitochondrial/energetic stress responses [274,275,276,277,278]. Evidence for these mechanisms derives predominantly from in vitro studies and animal models (including myocardial ischemia–reperfusion and infarction paradigms), whereas human data are heterogeneous and largely focused on surrogate cardiometabolic markers rather than hard cardiovascular endpoints. Interpretation is further complicated by strong formulation dependence driven by curcumin’s pharmacokinetic limitations (low aqueous solubility and rapid metabolism) [274,275,278,279]. The principal mechanisms, with types of evidence, are summarized below:

- Antioxidant and ROS-scavenging activity:

Curcuminoids and associated turmeric phenolics have been reported to reduce oxidative stress through direct scavenging and/or induction of endogenous antioxidant defenses, with studies describing decreased lipid peroxidation and preservation of antioxidant enzyme activity in experimental models [275,280,281]. In cardiac preclinical settings, turmeric/curcumin interventions have been associated with reduced ischemia–reperfusion injury, consistent with a contribution of redox-related mechanisms to observed protection [275,276,281].

- Anti-inflammatory signaling:

Curcumin also inhibits pro-inflammatory signaling, particularly NF-κB, and regulates inflammatory mediators and cytokines, contributing to reduced myocardial injury in animal models [275,277,282,283,284]. Clinical evidence for these effects, especially related to endothelial function in cardiovascular disease, is limited and necessitates targeted trials [279,285].

- Lipid-lowering effects:

Turmeric extracts demonstrate favorable effects on lipid metabolism in preclinical studies and some human trials, particularly in contexts like type-2 diabetes and metabolic syndrome, though variability in outcomes across studies suggests a need for larger, standardized trials [270,274,286]. Mechanistically, these lipid-modulatory actions may involve combined antioxidant, anti-inflammatory, and hepatic effects identified in prior research [268,274,284].

- Antithrombotic/anticoagulant and antiplatelet effects:

Turmeric and curcuminoids exhibit antithrombotic effects, inhibiting platelet aggregation and coagulation pathways in preclinical studies [273,286,287]. Despite promising findings, clinical evaluation concerning thrombotic outcomes remains sparse [279,287]. Given these preclinical antiplatelet/anticoagulant signals, interaction potential with antithrombotic medications should be explicitly addressed in clinical development and safety monitoring.

- Anti-apoptotic and anti-remodeling effects:

In animal models of myocardial ischemia–reperfusion and infarction, curcumin has been reported to reduce apoptosis-related readouts and infarct-size surrogates and to attenuate remodeling/fibrosis markers, suggesting that cell-survival and structural pathways may contribute alongside redox and inflammatory modulation in preclinical systems [274,275,276,284].

- Mitochondrial function and energy-metabolism modulation:

Turmeric extracts impact mitochondrial signaling pathways, preserving cellular bioenergetics and mitigating mitochondrial ROS generation [274,275,276]. However, there remains a lack of high-resolution human data concerning mitochondrial endpoints within cardiac tissues [278,279].

Curcumin has been explored as an adjunct to mitigate chemotherapeutic cardiotoxicity, with studies indicating potential attenuation of oxidative, inflammatory, and apoptotic pathways involved in cardiac damage [7,276]. While findings highlight potential roles for turmeric-derived agents as cardioprotective, translational development necessitates comprehensive safety and interaction studies [7,276,279].

Curcumin’s poor aqueous solubility, rapid metabolism, and low systemic bioavailability have motivated formulation strategies intended to increase exposure (e.g., adjuvants, lipid-based carriers, and other enhanced-delivery systems). While such approaches can increase circulating curcuminoid levels and have been associated with improved readouts in some experimental models, their clinical relevance requires PK/PD-informed evaluation and endpoint-driven trials using standardized products [276,278,285].

#### 3.9.4. General Conclusion

Overall, *Curcuma longa* and its curcuminoids, most prominently curcumin, have been reported to engage multiple pathways relevant to cardiovascular biology, including redox and inflammatory modulation, endothelial/NO-related signaling, lipid metabolism, platelet/coagulation-related processes, and mitochondrial stress responses. However, the strongest support for these mechanisms remains preclinical, whereas human evidence is limited, heterogeneous, and largely based on surrogate cardiometabolic markers; demonstration of reduced major cardiovascular clinical events is currently insufficient. Clinical translation is further constrained by pronounced pharmacokinetic limitations and strong formulation dependence, underscoring the need for standardized products and PK/PD-informed dosing [276,278,279,285].

Accordingly, future work should prioritize well-characterized extracts and enhanced-delivery formulations, mechanistic human studies using exposure biomarkers, and adequately powered randomized trials with harmonized endpoint sets that clearly distinguish validated surrogate outcomes from hard cardiovascular events [276,278,279,285]. Although turmeric is generally well-tolerated in dietary/traditional contexts, safety assessment in cardiovascular populations should explicitly address dose/formulation effects and potential interactions, particularly with antithrombotic therapies, before routine therapeutic recommendations can be supported [279,285,287].

### 3.10. Zingiber officinale *Roscoe* (Ginger)

#### 3.10.1. Historical Background

*Zingiber officinale* Roscoe (ginger, Zingiberaceae family) is a widely consumed culinary spice and traditional medicinal rhizome utilized in various traditional medical systems, including Traditional Chinese Medicine (TCM), Ayurveda, and other folk medicine practices across Asia and Europe. Historically, ginger has been used for gastrointestinal complaints and inflammatory conditions, and it is also described in ethnomedical sources for circulatory and cardiometabolic indications, which has motivated contemporary mechanistic and translational research [288,289,290]. In TCM, ginger is considered a “warming” agent and appears in multi-herb formulations like Sini decoction (SND), historically used for circulatory weakness and acute systemic stress states. These traditional attributions have motivated modern pharmacological interest in ginger-derived constituents in cardiovascular and cardiometabolic contexts [291,292]. Ethnopharmacological studies mention the application of fresh, dried, and processed ginger in postpartum care, for circulatory issues, and as an adjunct in multi-herb cardiac remedies, with the leaves of the plant also showing bioactive potential [289,293,294].

#### 3.10.2. Relevant Cardioprotective Phytocompounds

Cardiovascular-relevant phytochemicals in ginger rhizome are dominated by phenolic ketones, particularly gingerols (e.g., 6-gingerol) and their dehydration products shogaols (e.g., 6-shogaol), alongside related metabolites (paradols, zingerone) and a volatile terpene fraction (e.g., zingiberene, β-sesquiphellandrene) [288,293,295,296]. The rhizome typically has a volatile oil content of approximately 2–3% of its dry weight, along with fatty/oil fractions totaling around 3–6%. Moreover, the gingerol:shogaol ratio alters significantly based on processing methods (fresh vs. dried/heated), influencing its antioxidant, anti-inflammatory, and metabolic activities [288,289,293]. Although the rhizome is primarily used in cardiovascular research, ginger leaves exhibit diverse antioxidant properties and may serve as alternative sources for phytochemical exploration [288,294].

#### 3.10.3. Mechanisms of Cardioprotective Effects and Medicinal Benefits

The principal mechanisms, with types of evidence, are summarized below:

- Antioxidant and ROS-scavenging activity:

Ginger and its constituents have been reported to exhibit radical-scavenging activity and to modulate endogenous antioxidant defenses in biochemical assays, cell cultures, and animal models, potentially mitigating oxidative processes implicated in atherogenesis and ischemia–reperfusion injury [288,295,297,298]. Experimental studies describe decreased lipid peroxidation and changes in antioxidant enzyme activities after ginger/constituent exposure in metabolic-stress models, consistent with a redox-related contribution to vascular and myocardial resilience in preclinical settings [293,295,299,300].

- Anti-inflammatory signaling (NF-κB, cytokine modulation):

Evidence from in vitro and animal studies suggests that ginger bioactives can attenuate inflammatory pathways relevant to cardiovascular disease biology, including reported effects on NF-κB-related signaling, COX-2/iNOS-associated responses, and pro-inflammatory cytokine readouts [288,297,299,301]. In animal models, gingerols and shogaols have been associated with reduced systemic/vascular inflammatory markers alongside improvements in vascular function and metabolic parameters, supporting anti-atherosclerotic plausibility in experimental settings [299,300,302,303].

- Endothelial protection and nitric oxide (NO) regulation:

Endothelium-dependent vasorelaxation due to ginger extracts has been observed in ex vivo studies of coronary arteries and systemic vascular models. These protective effects have been associated with increased endothelial NO bioavailability and the activation of soluble guanylyl cyclase signaling [288,303,304]. In hypercholesterolemic animal studies, ginger has been found to reduce arginase activity and oxidative stress, mechanisms that sustain endothelial L-arginine availability and NO signaling, potentially supporting endothelial function and mitigating dysfunction central to atherothrombosis [288,299,300].

- Lipid-lowering and anti-atherosclerotic effects:

A growing body of preclinical evidence highlights that ginger and specific constituents can improve lipid profiles by reducing total cholesterol, LDL, and triglycerides, while sometimes modulating HDL levels. The proposed mechanisms involve regulation of lipid metabolism through peroxisome proliferator-activated receptors (PPARs), adenosine monophosphate-activated protein kinase (AMPK) activation, and inhibition of intestinal lipid absorption [288,299,301,302]. Human evidence is more limited and largely surrogate-based: for example, a small, short-duration randomized trial (30 days) reported changes in blood lipids and glycemic indices in hypertensive older women, providing preliminary clinical support that requires replication with standardized preparations and longer follow-up [299,303,305].

- Anti-apoptotic and anti-fibrotic pathways:

The regulation of cell death pathways by ginger is context-dependent. In oncology models, pro-apoptotic effects are leveraged. Conversely, studies related to cardiometabolic conditions and cardiac injury show that ginger can mitigate ischemia–reperfusion-associated apoptotic signaling and reduce fibrotic remodeling in experimental setups [288,295,303].

- Mitochondrial protection and energy-metabolism modulation:

Ginger and compounds such as 6-gingerol have been linked in experimental models to activation of energy-sensing pathways (e.g., AMPK) and modulation of PPAR-related signaling, with reported effects on fatty-acid oxidation and insulin sensitivity. Whether these changes translate into direct myocardial mitochondrial protection in humans remains insufficiently characterized [288,299,301,302].

- Antiplatelet and antithrombotic activity:

In vitro studies report that ginger extracts inhibit platelet aggregation and reduce thromboxane B2 formation, suggesting potential antithrombotic roles pertinent to preventing occlusive cardiovascular events. However, these antiplatelet effects necessitate caution as they could interact with anticoagulant therapies for patients with cardiovascular disease [288,303,304].

Overall, the evidence base is dominated by mechanistic in vitro studies and animal models reporting effects on vascular reactivity, redox/inflammatory readouts, lipid and glycemic parameters, and ischemic-injury surrogates [295,297,299,300,301,302,304]. Human data are comparatively modest and often short-term, focusing mainly on cardiometabolic risk markers rather than hard cardiovascular outcomes, with additional clinical literature addressing non-cardiac indications [288,303,305,306,307]. Key translational barriers include strong preparation and processing dependence (fresh vs. dried/heated materials; powders vs. extracts), variability in gingerol/shogaol profiles, limited pharmacokinetic/PK–PD data, and between-study heterogeneity, all of which complicate dose–response interpretation and definitive efficacy claims in cardiovascular contexts [288,299].

#### 3.10.4. General Conclusion

*Zingiber officinale* is a chemically complex botanical rich in gingerols/shogaols (e.g., 6-gingerol, 6-shogaol), related phenolic metabolites (e.g., zingerone), and a volatile terpene fraction. Across experimental systems, ginger preparations have been reported to modulate redox and inflammatory readouts, influence endothelial/vasomotor signaling, and affect cardiometabolic pathways linked to lipid and glucose handling (often discussed in relation to AMPK/PPAR-associated signaling), with additional preclinical reports of mitochondrial- and platelet-related effects [288,293,299,303,304]. However, the evidence base remains predominantly preclinical, and available human studies are limited in size and duration and largely report changes in surrogate cardiometabolic risk markers rather than hard cardiovascular outcomes.

From a translational and safety perspective, tolerability is generally acceptable in many short-term studies, but preparation- and dose-dependence, potential antiplatelet activity, and herb–drug interaction potential (particularly with antithrombotic and cardiovascular medications) warrant caution and systematic evaluation in relevant patient populations [288,303,304]. Future research should prioritize marker-based standardization (including gingerol:shogaol profiling and processing documentation), PK/PD-informed dosing and exposure biomarkers, rigorous interaction and bleeding-risk assessment, and adequately powered randomized trials with harmonized endpoint sets and mechanistic human readouts (e.g., endothelial function) to clarify clinical utility [288,291,299,303].

**Figure 2:**
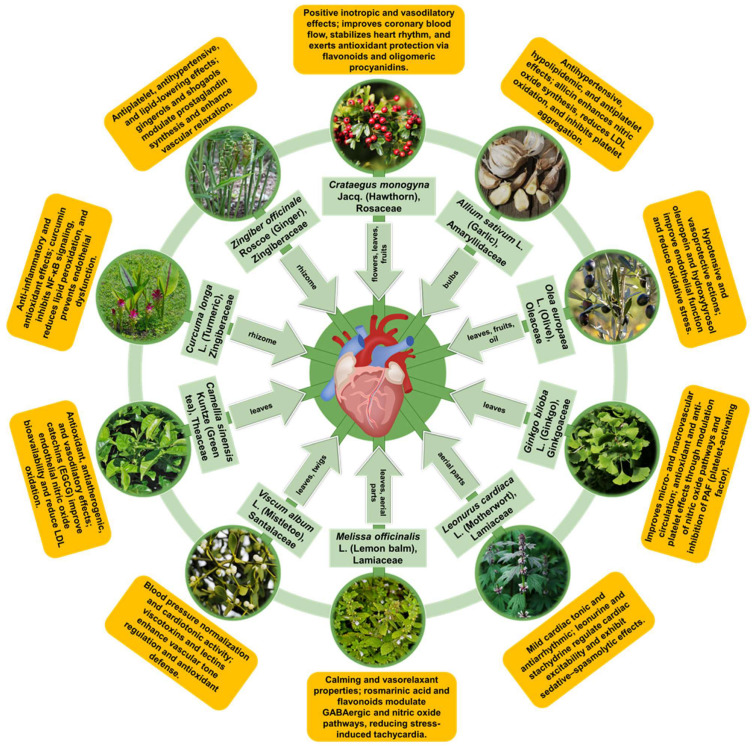
Ten medicinal plants with cardioprotective properties, their botanical families, used parts, and main cardiovascular effects and mechanisms of action: *Crataegus monogyna* (https://www.alamy.com/stock-photo-hawthorn-or-may-tree-berries-crataegus-monogyna-rosaceae-25574046.html, accessed on 1 December 2025), *Allium sativum* (https://www.alamy.com/allium-sativum-garlic-image611332157.html?imageid=DB4D97D2-FC14-4E47-A812-23091E24A4DD&pn=5&searchId=1bb5257ee378362cd03b04e52d6022b3&searchtype=0, accessed on 1 December 2025), *Olea europaea* (https://www.alamy.com/olea-europaea-black-olives-on-the-branch-image336193729.html?imageid=85EBD50E-B50E-4CCF-A87A-1ACBF9B92004&pn=1&searchId=6c86496276aed364a66fadf30834ea79&searchtype=0, accessed on 1 December 2025), *Ginkgo biloba* (https://www.alamy.com/stock-photo-leaves-of-the-maidenhair-tree-ginkgo-biloba-ginkgoaceae-south-east-19860602.html?imageid=189E17B8-2960-45F5-864F-45E1B953B8E1&pn=1&searchId=ba1a9a84fb400564bd96f470e3766967&searchtype=0, accessed on 1 December 2025), *Leonurus cardiaca* (https://www.alamy.com/stock-photo-herzgespann-echtes-herzgespann-leonurus-cardiaca-86380230.html?imageid=5DA4115C-845D-414F-975F-DE59B7017297&pn=1&searchId=cf9136963a27b1aba7336fe579bc21ad&searchtype=0, accessed on 1 December 2025), *Melissa officinalis* (https://www.alamy.com/stock-photo-lemon-balm-garden-balm-melissa-officinalis-blooming-germany-76107085.html, accessed on 1 December 2025), *Viscum album* (https://www.alamy.com/stock-photo-mistletoe-berries-viscum-album-on-female-plant-growing-in-an-apple-54468239.html?imageid=325DB7A5-387B-4EB1-8733-4027E96C1A47&pn=1&searchId=1e430a64d0bc6063b2dea6922346fdf9&searchtype=0, accessed on 1 December 2025), *Camellia sinensis* (https://www.alamy.com/tea-bush-camellia-sinensis-young-leaves-nerada-tea-plantation-malanda-image61900781.html?imageid=32ECA355-DE71-44BF-B995-8FE4D6F29846&pn=1&searchId=0d95ff7ecff893072c98a1c754bab939&searchtype=0, accessed on 1 December 2025), *Curcuma longa* (https://www.alamy.com/turmeric-or-curcumin-plant-curcuma-longa-of-the-ginger-family-image395631701.html?imageid=C197D5F2-D7FA-4DD9-A1DD-C1F535928A37&pn=1&searchId=d614d188a0418ddc03b8845eb8e20b9e&searchtype=0, accessed on 1 December 2025), *Zingiber officinale* (https://www.alamy.com/ginger-flower-this-is-true-flower-of-edible-ginger-plant-zingiber-officinalis-image596823628.html?imageid=BE8C5085-5B80-44BE-9851-4AB818997C39&pn=1&searchId=b364abef0dfe7028730e09f9c50deb45&searchtype=0, accessed on 1 December 2025).

The cardioprotective potential of medicinal plants arises from their ability to modulate multiple molecular pathways involved in oxidative stress, endothelial function, lipid metabolism, and cardiac contractility. Within this framework, Figure 2 provides a concise graphical summary of Section 3, depicting the main cardiovascular effects, mechanisms of action, and plant parts used from ten well-documented species with demonstrated cardioprotective activity.

## 4. Limitations, Challenges, and Safety Issues

The cardiac benefits attributed to plant-derived bioactive compounds have been demonstrated in various preclinical models and in some early clinical models through a series of mechanisms relevant to cardioprotection, including oxidative stress modulation, anti-inflammatory effects, antiatherosclerotic actions, improvements in endothelial/vascular function, and attenuation of cardiac remodeling and ischemia–reperfusion injury [308,309,310]. Although these avenues are promising, translating these discoveries into robust clinical applications remains a challenge, and safety considerations are essential in evaluating their therapeutic potential [308,309,310,311].

Many studies evaluating cardioprotective phytochemicals are preclinical in nature, with heterogeneous designs and objectives and a tendency toward short study durations, which may limit the strength of evidence regarding long-term clinical benefits [311,312,313]. The diversity of models and variability of outcome measures complicate synthesis across studies and interpretation of results, while also hindering regulatory evaluation, highlighting the need for standardized research practices and more robust clinical trials [311,312]. Furthermore, although preclinical data are encouraging, translating these effects into meaningful results in humans requires randomized, well-documented studies with appropriate objectives and a longer follow-up period [309,311,313]. These translational gaps are found in discoveries about plant-derived antioxidants and cardioprotective phytochemicals, where preclinical success has not consistently culminated in proven clinical efficacy [309,311].

Beyond the signals of efficacy, there is a recognized lack of standardized information on optimal dosing, pharmacokinetics, exposure–response relationships, and long-term safety in the clinical setting, which may undermine the reproducibility and replicability of the reported benefits [307,312]. Contemporary guidelines highlight uncertainties regarding minimum active concentrations, dosing frequency, and comprehensive toxicity assessment, all of which hinder reproducible translation from the laboratory to the patient’s bedside [307,312,314]. In addition, complex plant matrices often produce variable levels of constituents, which pose an additional challenge to generalizability and acceptance by regulatory authorities [311,315].

A pervasive limitation of plant-derived cardioprotective compounds is suboptimal bioavailability and pharmacokinetic profiles that limit therapeutic exposure at target sites. Poor absorption, rapid metabolism, and limited tissue distribution are repeatedly cited obstacles to achieving reliable cardioprotective effects in humans, necessitating the development of improved delivery systems and formulation strategies [307,311]. For example, analyses of specific compounds and general nutraceuticals highlight ADME constraints that complicate dose optimization and therapeutic consistency in the clinical setting [309,316,317,318]. A representative example is curcumin, where poor aqueous solubility and extensive first-pass metabolism yield low and highly variable systemic exposure with conventional powders, motivating enhanced-delivery formulations (e.g., lipid-based carriers or adjuvant-based approaches) to increase circulating levels; however, improved exposure also raises the need for PK/PD-guided dose selection and safety monitoring in target cardiovascular populations. Several plant-derived candidate substances show promise in preclinical models but do not achieve clinically relevant exposure without advanced delivery approaches. This is further underscored by the demands for scalable extraction methods, stabilization, and solubility improvements to enable consistent dosing and meaningful pharmacokinetics in humans [309,311,316]. The need for better bioavailability is often associated with requests for integrative studies that combine pharmacokinetics with pharmacodynamics to establish exposure–response relationships in humans [309,318].

Safety concerns are crucial for promoting the clinical use of plant-derived cardioprotective compounds. Comprehensive toxicological evaluation, including potential organ toxicity, drug interactions, and long-term safety, is often lacking or insufficient in early-phase studies, raising questions about human safety and the risk-benefit ratio for chronic cardiovascular indications [319,320]. For example, toxicological data on *Moringa oleifera* leaf extract document potential adverse changes at high doses in animal models, highlighting the importance of safety assessments regarding doses and the need for rigorous clinical toxicology before human use regarding cardioprotection [320]. Another example, rare but clinically significant hepatotoxicity has been reported with concentrated green tea (*Camellia sinensis*) extracts in supplement contexts, underscoring the importance of dose, formulation, and exposure-dependent safety assessment. This safety signal has prompted region-specific risk-management actions in Europe, including restrictions and labeling requirements for products delivering high (−)-epigallocatechin-3-gallate (EGCG) doses, illustrating how safety concerns can shape regulatory decisions for botanicals intended for chronic use [240,241,243,245,246,247]. Despite preclinical cardioprotective signals, safety issues such as toxicity, interactions between herbal medicines and drugs, and unpredictable pharmacokinetics must be addressed to ensure the safe clinical application of phytochemicals in cardiovascular indications; these issues are particularly relevant when considering polypharmacy in cardiovascular patients and potential interactions with standard therapies [318,319]. Clinically relevant interaction concerns are most commonly raised for botanicals with antiplatelet/anticoagulant activity (e.g., *Ginkgo biloba*- or *Allium sativum*-based products) when co-administered with antithrombotic therapies, reinforcing the need for proactive interaction assessment and monitoring strategies in cardiovascular polypharmacy [106,107,108,163,174,175]. In silico toxicity profiling and early safety screening tools are valuable for flagging potential issues at an earlier preclinical stage, but they cannot replace empirical toxicological data from well-designed studies [314].

A major challenge concerning clinical translation is the lack of standardization of plant materials, extraction procedures, and analytical systems. Variability in phytochemical content between plant sources and preparations undermines the comparability and reproducibility of results, complicating dose standardization and safety profiling [311,315,321]. For example, analyses of phytochemical use in the cardiovascular context highlight the need for rigorous analytical characterization of active constituents and standardized dosing regimens to enable reproducible clinical studies and regulatory evaluation [312,315,321]. The absence of universally accepted guidelines for the presentation and evaluation of phytochemical-based cardiovascular therapies further complicates regulatory approvals, reinforcing the requirement for rigorous methodologies and transparent reporting of dose, exposure, toxicity, and evaluation criteria in preclinical and clinical studies [312,314].

To move plant-derived cardioprotectants from largely preclinical signals toward clinically testable interventions, several practical solutions can be implemented. First, standardization should be approached as “fit-for-purpose” quality control: detailed reporting of plant material (species/authentication, plant part, harvest/processing), batch-to-batch chemical fingerprinting (e.g., HPLC/LC-MS profiles), and marker-based quantification of key constituents to enable reproducibility and meaningful dose comparisons. Second, bioavailability constraints should be addressed using formulation strategies (e.g., lipid-based carriers, phytosomes, nanoemulsions, or stabilized extracts), but only in parallel with PK/PD-guided dose selection, exposure biomarkers, and explicit definition of the circulating moieties expected to drive biological effects. Third, herb–drug interaction risk should be assessed proactively through a tiered workflow: in vitro screening for CYP/UGT/transporters and platelet/coagulation effects, followed by mechanistic modeling and targeted clinical interaction studies in relevant cardiovascular polypharmacy settings. Fourth, clinical trial design should prioritize (i) standardized products with verified composition and stability, (ii) prespecified, harmonized endpoint sets that distinguish validated surrogate markers from hard cardiovascular outcomes, (iii) adequate duration and statistical power, and (iv) rigorous adverse-event capture and pharmacovigilance. Collectively, these measures provide a pragmatic translational pathway to reduce heterogeneity, improve interpretability, and enable regulatory-grade evaluation of efficacy and safety for cardioprotective phytotherapy [311,312,314,315,321].

Regulatory expectations also vary by region. In the European Union, certain botanical products may fall under the Traditional Herbal Medicinal Products framework and associated monographs/quality requirements, which can facilitate harmonized assessment but also impose specific standards for quality, consistency, and documentation. In contrast, in the United States, many products are marketed as dietary supplements under a distinct regulatory paradigm, where premarket requirements differ from those for medicinal products. Consequently, evidence thresholds, quality documentation, and claims management can vary substantially across jurisdictions [322,323,324].

There is a broad consensus that, despite encouraging preclinical data, the translation of plant-derived cardioprotective agents into clinically validated therapies requires more rigorous clinical studies in humans, standardized preparations, and robust safety data. Current evidence is insufficient to draw definitive conclusions about efficacy in humans, requiring well-controlled clinical trials with standardized formulations, long-term safety monitoring, and clear regulatory pathways [311,312,313,314].

In silico toxicity prediction and data sharing initiatives illustrate the importance of early identification of safety issues regarding cardiotoxicity and other adverse effects, but also highlight that even high-quality predictive models do not eliminate the need for comprehensive preclinical and clinical safety assessment before human use [314,325,326]. It is recommended to integrate computational toxicology with in vivo and in vitro data to better anticipate adverse outcomes associated with plant-derived cardioprotective compounds [314].

Beyond conventional pharmacology, emerging omics technologies can improve the reproducibility and translational interpretability of cardioprotective phytotherapy. Untargeted metabolomics/chemometrics can generate robust phytochemical fingerprints for batch control and detection of formulation variability, while transcriptomic/proteomic and single-cell approaches may provide mechanistic signatures and clinically relevant biomarkers to align preclinical models with human pathophysiology. In parallel, machine learning and neural network-based methods can integrate multi-omic, pharmacokinetic, and clinical datasets to (i) model exposure–response relationships and optimize dosing strategies, (ii) predict safety liabilities and interactions (e.g., via transporter/enzyme liability and real-world pharmacovigilance data), and (iii) prioritize mechanistic hypotheses from systems/network pharmacology for experimental validation. Importantly, these approaches require high-quality standardized inputs, transparent reporting, and regulatory-grade validation before they can meaningfully guide clinical decision-making.

## 5. Conclusions and Future Directions

Plant-derived bioactive compounds represent a scientifically grounded and mechanistically diverse source of cardioprotective agents. Across experimental and translational research, phytochemicals such as flavonoids, phenolic acids, organosulfur compounds, terpenoids, and secoiridoids have demonstrated the ability to modulate key pathogenic mechanisms involved in cardiovascular disease, including oxidative stress, inflammation, endothelial dysfunction, mitochondrial instability, and apoptosis. Evidence from preclinical models consistently indicates that these compounds can attenuate ischemia/reperfusion injury, preserve myocardial structure and function, and improve hemodynamic and metabolic parameters.

Among the best-documented medicinal plants, *Crataegus monogyna*, *Allium sativum*, *Olea europaea*, *Ginkgo biloba*, *Leonurus cardiaca*, *Melissa officinalis*, *Curcuma longa*, and *Zingiber officinale* exhibit complementary cardioprotective actions mediated by complex phytochemical profiles acting through multiple molecular targets. Clinical studies provide promising, yet heterogeneous, evidence of benefit in improving endothelial function, blood pressure, lipid metabolism, and oxidative biomarkers.

From a clinical-context perspective, the available evidence (predominantly surrogate outcomes and heterogeneous human trials) supports an indication-oriented prioritization for future, adequately powered studies rather than broad, non-specific “cardioprotection” claims. For hypertension and vascular dysfunction, the most consistent human signals across the plants reviewed relate to blood pressure and endothelial function, motivating standardized interventions with garlic preparations (formulation-dependent OSC exposure) and phenolic-enriched olive products, with prespecified endpoints such as ambulatory blood pressure and flow-mediated dilation. For heart failure and functional limitation, standardized hawthorn preparations (e.g., WS^®^1442) represent a leading candidate for adjunctive evaluation, with emphasis on reproducible dosing, exercise tolerance, symptom scores, and safety in polypharmacy settings. For atherosclerotic dyslipidemia and cardiometabolic risk, olive phenolics and green-tea catechins (and, in selected metabolic contexts, ginger/curcumin formulations) are best positioned for trials targeting lipids/remnant risk, inflammatory biomarkers, and vascular function, while explicitly acknowledging modest effect sizes and formulation-driven variability. For arrhythmia-related symptoms (palpitations, autonomic dysregulation), motherwort and lemon balm are supported mainly by traditional use and preclinical/ex vivo electrophysiology, warranting hypothesis-driven clinical studies with ECG/Holter endpoints, autonomic markers, and careful interaction monitoring. Finally, botanicals with platelet-modulating properties (e.g., *Ginkgo* preparations) require a safety-first framework in cardiovascular populations, prioritizing interaction studies with antithrombotic drugs before broader efficacy testing. This indication-oriented framing clarifies where the current evidence base is most actionable and where it remains primarily preclinical, thereby improving clinical interpretability and trial design.

Despite this growing body of evidence, translation into clinical practice remains limited by variability in extract standardization, bioavailability, and study design. Future research should focus on defining standardized phytochemical compositions, elucidating pharmacokinetic and pharmacodynamic interactions with conventional cardiovascular drugs, and validating efficacy through well-designed randomized controlled trials with mechanistic and clinical endpoints. The integration of phytochemical cardioprotective strategies into modern cardiovascular medicine requires a rigorous scientific framework that bridges molecular mechanisms, safety, and clinical outcomes.

Advancing the clinical utility of plant-derived cardioprotective agents will require a multidisciplinary strategy that integrates pharmacognosy, molecular pharmacology, and clinical research. Priority directions include improving bioavailability and stability through novel formulations (e.g., nanoencapsulation, liposomal, or phytosomal delivery systems), identifying reliable biomarkers of exposure and pharmacodynamic response, and employing omics-based approaches to elucidate complex molecular networks affected by phytochemicals. Standardization of extracts and rigorous quality control are essential to ensure reproducibility and translational comparability across studies.

Furthermore, combinatorial strategies that pair phytochemicals with conventional cardioprotective drugs hold promise for the synergistic enhancement of efficacy and safety, particularly in conditions characterized by residual cardiovascular risk. Comprehensive clinical programs should incorporate systems biology, pharmacokinetic modeling, and network pharmacology to clarify dose–response relationships and potential interactions. Ultimately, integrating evidence-based phytotherapy into cardiovascular prevention and treatment paradigms could contribute to more personalized and mechanistically informed interventions, bridging traditional knowledge and modern molecular medicine.

## Data Availability

There are no additional data to be published.

## Abbreviations

The following abbreviations are used in this manuscript:

Akt	Protein kinase B
AP-1	Activator protein-1
Bcl-2	B-cell lymphoma 2
CAT	Catalase
CK	Creatine kinase
COX-2	Cyclooxygenase-2
CVD	Cardiovascular disease
DADS	Diallyl disulfide
DATS	Diallyl trisulfide
DAS	Diallyl sulfide
EGb 761	Standardized *Ginkgo biloba* extract (24% flavone glycosides, 6% terpene lactones)
EGCG	Epigallocatechin gallate
ERK	Extracellular signal-regulated kinase
EVOO	Extra virgin olive oil
GSH	Glutathione
GSK-3β	Glycogen synthase kinase-3 beta
H_2_S	Hydrogen sulfide
HKII	Hexokinase II
HO-1	Heme oxygenase-1
HT	Hydroxytyrosol
ICAM-1	Intercellular adhesion molecule-1
IL	Interleukin
iNOS	Inducible nitric oxide synthase
I/R	Ischemia/reperfusion
IPC	Ischemic preconditioning
KATP	ATP-sensitive potassium channel
LDH	Lactate dehydrogenase
MAPK	Mitogen-activated protein kinase
MCP-1	Monocyte chemoattractant protein-1
mPTP	Mitochondrial permeability transition pore
MQC	Mitochondrial quality control
NF-κB	Nuclear factor kappa-light-chain-enhancer of activated B cells
NO	Nitric oxide
Nrf2	Nuclear factor erythroid 2-related factor 2
OSCs	Organosulfur compounds
PAF	Platelet-activating factor
PGC-1α	Peroxisome proliferator-activated receptor gamma coactivator 1-alpha
PI3K	Phosphatidylinositol 3-kinase
PKC	Protein kinase C
RISK	Reperfusion injury salvage kinase pathway
ROS	Reactive oxygen species
SAC	S-allylcysteine
SAMC	S-allylmercaptocysteine
SIRT1	Sirtuin 1
SOD	Superoxide dismutase
SREBP-1	Sterol regulatory element-binding protein 1
TNF-α	Tumor necrosis factor alpha
TXNIP	Thioredoxin-interacting protein
VCAM-1	Vascular cell adhesion molecule-1
WS^®^1442	Standardized *Crataegus* extract (hawthorn)
